# Strategies toward protecting group-free glycosylation through selective activation of the anomeric center

**DOI:** 10.3762/bjoc.13.123

**Published:** 2017-06-27

**Authors:** A Michael Downey, Michal Hocek

**Affiliations:** 1 Institute of Organic Chemistry and Biochemistry, Czech Academy of Sciences, Flemingovo nam. 2, 16610 Prague 6, Czech Republic; 2 Department of Organic Chemistry, Faculty of Science, Charles University in Prague, 12843 Prague 2, Czech Republic

**Keywords:** glycosides, glycosylation, oligosaccharides, protecting groups

## Abstract

Glycosylation is an immensely important biological process and one that is highly controlled and very efficient in nature. However, in a chemical laboratory the process is much more challenging and usually requires the extensive use of protecting groups to squelch reactivity at undesired reactive moieties. Nonetheless, by taking advantage of the differential reactivity of the anomeric center, a selective activation at this position is possible. As a result, protecting group-free strategies to effect glycosylations are available thanks to the tremendous efforts of many research groups. In this review, we showcase the methods available for the selective activation of the anomeric center on the glycosyl donor and the mechanisms by which the glycosylation reactions take place to illustrate the power these techniques.

## Review

### 1 Introduction

The glycosylation reaction is of extreme importance in nature as it is possibly the most prevalent post-translational modification and thus has implications in a tremendous number of biological processes, including diseases [1]. More expedient chemical and enzymatic methods to access glycosides is an ongoing area of research and one that could have implications that extend far beyond a synthetic chemist’s laboratory [2–4]. Glycosylation is a coupling reaction that takes place at the anomeric position (C1–OH) of a saccharide, termed donor, and another molecule, termed the acceptor, with the product of the reaction termed glycoside. Examples of acceptor molecules in nature are other saccharides to form oligosaccharides, nucleobases to form nucleosides, and amino acid side chains to form glycoproteins. The donor is the electrophile in the reaction and, therefore, when attempting glycosylation, generally the other reactive (nucleophilic) groups on the saccharide must be protected to prevent reaction with itself. The accepting molecule is nucleophilic and very often complex as well, and, thus, must also be protected to squelch reactivity at undesired reactive groups. As a result, in synthetic chemistry, this process of glycosylation is very often cumbersome and can involve the use of highly toxic reagents [5–8].

**Scheme 1:**
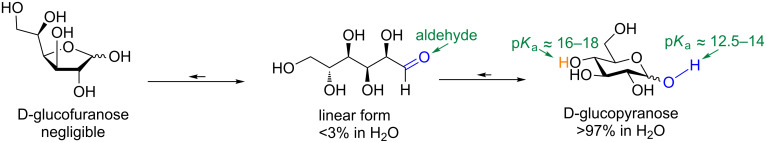
Solution-state conformations of D-glucose.

The idealized scenario would be a glycosylation strategy that can occur in the complete absence of protecting groups under mild, neutral conditions. The linchpin for protecting-group-free glycosylation is an exploitation of the differential reactivity of the anomeric center. Two key features of the anomeric center provide this possibility. Firstly, the anomeric position of all unprotected monosaccharides is a reducing end (i.e., in equilibrium as an aldehyde or ketone) making this center more electrophilic (Scheme 1) [3]. Secondly, the p*K*_a_ value of the anomeric OH group (glucose p*K*_a_ ≈ 12.5 [9] or 14 [10]) is several orders of magnitude lower than for the other hydroxy groups (p*K*_a_ ≈ 16–18) [9–10] so a careful selection of the base should allow for the selective deprotonation of this hydroxy group over the others. This selective creation of a better nucleophile in the presence of the other protonated hydroxy groups can be regarded as an umpolung process.

Despite this seeming difficulty, some protecting-group-free strategies to synthesize glycosides and nucleosides have been developed. We highlight some classical enzymatic and synthetic strategies below before discussing chemical strategies that have been developed or rediscovered chiefly since the beginning of this century. Special emphasis is drawn to the mechanisms by which the glycosylations take place. As demonstrated by the increasing number of papers published recently, it is clear this scientific field is rapidly expanding. During the preparation of this article a very nice thorough review of some aspects of protecting-group-free glycosylation reactions has been published by Jensen, Thygesen, and co-workers [11]. While their review is a comprehensive overview of many diverse glycosylation strategies, we offer a more detailed account of the methods developed in the last two decades with the main focus on the processes developed for the selective activation of the anomeric center, hence circumventing the requirement to protect the other nucleophiles on the donor molecule.

### 2 Classical glycosylation strategies

#### 2.1 Enzymatic strategies

Chemoenzymatic glycosylation largely involves two classes of enzymes: glycosynthases engineered for the synthesis of oligosaccharides and glycosyltransferases for the synthesis of oligosaccharides, glycoproteins, and nucleosides, both, natural and synthetic. The advantages of utilizing this approach are obvious as the reactions take place using unprotected saccharide donors and acceptor molecules. However, these methods are not without substantial challenges. A brief overview of these two methods is given in this section.

**Scheme 2:**
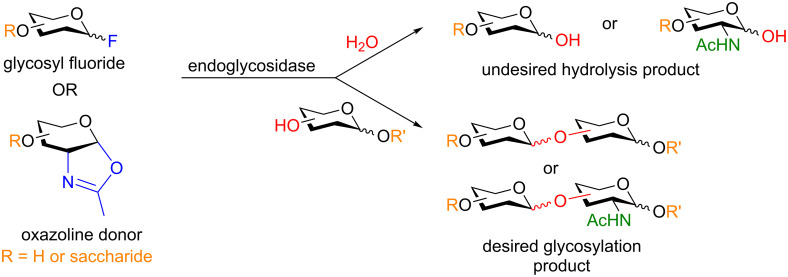
Enzymatic synthesis of oligosaccharides.

**2.1.1 Glycosynthases:** Glycosynthases catalyze the formation of a glycosidic bond between two saccharide moieties and were evolved from naturally occurring glycosidase enzymes, which, in fact, catalyze the hydrolysis (i.e., the reverse process) of glycosyl bonds [12]. Classical approaches in the transglycosylation of saccharides to form larger oligosaccharides utilize an endoglycosidase that couples a donor and an acceptor in situ to provide the lengthened oligosaccharide [13–14]. The primary challenge in transglycosylation strategies has always been the competing hydrolysis reaction, which is thermodynamically favored. However, due to incredible efforts in the field this problem can be mostly circumvented by the selection of the appropriate mutant through directed evolution [12] and advancements in donor design being the key players [15]. The two most successful saccharide donors to date are 1-fluoroglycosides or oxazoline derivatives (Scheme 2). In addition to the aforementioned enzymes, thioglycoligases and thioglycosynthases have also been developed for the synthesis of thioglycosides as reviewed by Withers et al. [12].

**Scheme 3:**
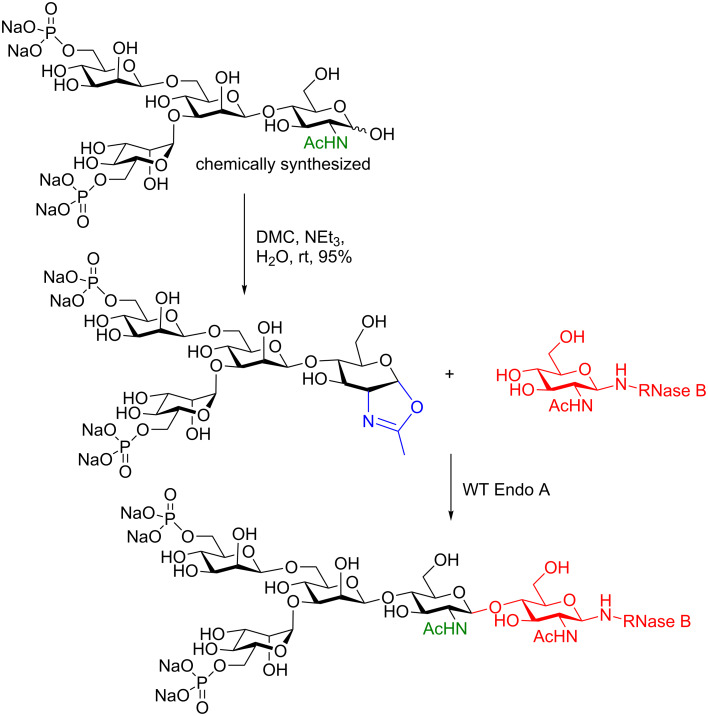
Enzymatic synthesis of a phosphorylated glycoprotein containing a mannose-6-phosphate (M6P)-terminated N-glycan. DMC = 2-chloro-1,3-dimethylimidazolinium chloride.

An elegant example of this powerful methodology has been reported from the Fairbanks group [16] recently. They synthesized a phosphorylated glycoprotein containing a mannose-6-phosphate (M6P)-terminated *N*-glycan (Scheme 3). Their work combined the chemical synthesis of a phosphotetrasaccharide with the enzymatic ligation of an oxazoline donor and commercially available RNase B protein (with the glycans curtailed to a single GlcNAc moiety [17]) as the acceptor [16]. The beauty of this work extends beyond the enzymatic glycosylation reaction. We point out that the oxazoline functionality was installed chemically in the complete absence of protecting groups in an aqueous environment using 2-chloro-1,3-dimethylimidazolinium chloride (DMC) and a mild amine base (NEt_3_) (see chapter 4.1).

**Scheme 4:**
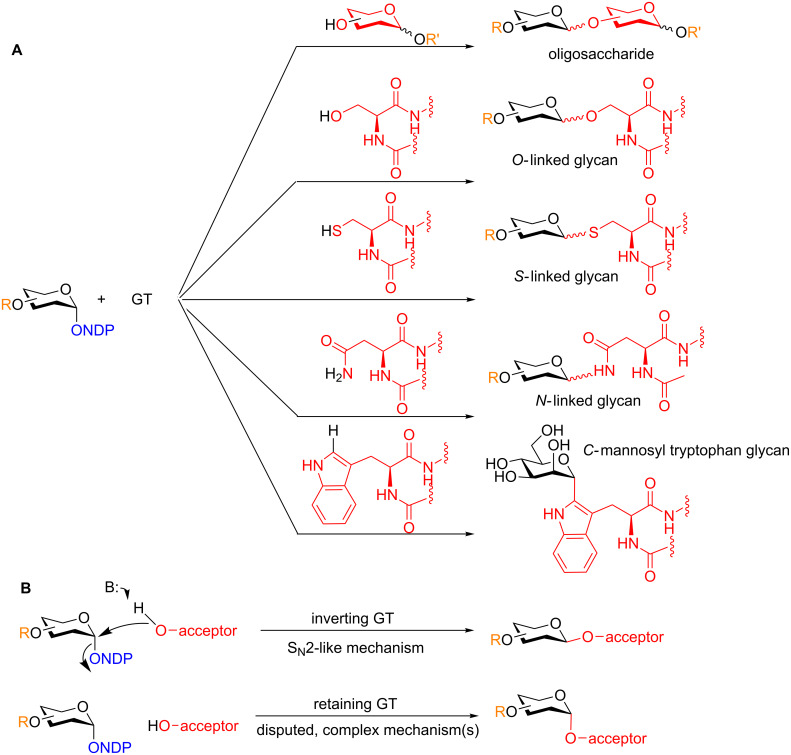
A) Selected GTs-mediated syntheses of oligosaccharides and other biologically active glycosides. B) Inverting and retaining GTs. NDP = nucleotide diphosphate.

**2.1.2 Glycosyltransferases:** Glycosyltransferases (GTs) catalyze the transfer of a carbohydrate from an activated nucleotide saccharide donor to a nucleophilic glycosyl acceptor to provide *O*-, *N*-, *S*- [18] and even *C*-linked [19] glycosides (Scheme 4A). The GTs can be divided into two subclasses, inverting and retaining, depending on their mechanism of action [20]. Inverting GTs provide glycosides whereby the acceptor is glycosylated with the opposite stereochemistry at the anomeric position to the donor in an S_N_2-like mechanism that is reasonably well understood. Retaining GTs provide glycosides with the same stereochemistry at the anomeric position as the donor. However, the mechanism(s) is less well understood and is still subject of much debate (Scheme 4B) [21]. The utility of these enzymes is very clear and even extends beyond glycobiology. They are applicable to natural product synthesis as the aglycone of a natural product glycoside can be forged to the saccharide component using either a natural or engineered GT [22]. On the other hand, the disadvantage is the need to synthesize NDP-sugars as substrates for the GT which is typically a multistep laborious process.

**Scheme 5:**
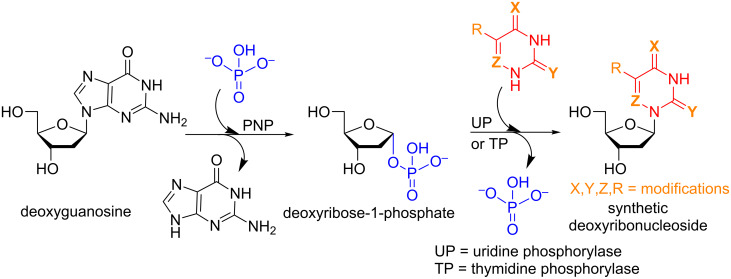
Enzymatic synthesis of nucleosides.

Another particularly interesting application of glycosyltransferases is the chemoenzymatic synthesis of nucleosides. This is an incredibly powerful tool, as these enzymes can be utilized in drug design and hence has value to the medical community as well. These enzymes also are generally regioselective for the position 9 of purines and the position 1 of pyrimidines which is a persisting challenge in the chemical synthesis of biologically active nucleosides [23–24]. The enzymes typically employed for these purposes are nucleoside phosphorylases (NPs) or nucleoside deoxyribosyltransferases (NDTs) in the presence of inorganic phosphate in a tandem enzymatic process [25]. In Scheme 5 we highlight a very recent example of this methodology for the synthesis of modified pyrimidine nucleosides using *E. coli* NPs [26].

#### 2.2 Synthetic strategies

**Scheme 6:**
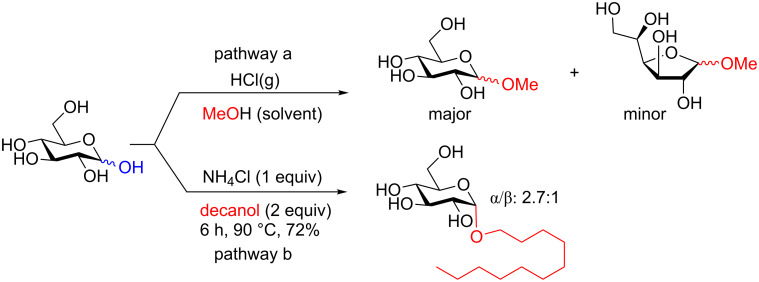
Fischer glycosylation strategies.

The classical protecting-group-free (pre-2000) synthetic strategies are dated back to well over 100 years with the discovery of the Fischer glycosylation (Scheme 6) [27–28]. Methanol can be glycosylated with D-glucose in the presence of HCl to provide the methyl glycoside (pathway a, Scheme 6). The reaction proceeds chemoselective at the anomeric position. More recent examples typically use Lewis acids [29–34] or microwave irradiation [35–36] to accelerate the reaction. However, shortcomings still include the need to use stoichiometric or excessive quantities of the often toxic acid as well as long reaction times, high temperature, and almost a complete lack of stereochemical control [37–38]. We highlight one recent interesting example from 2013 where ammonium chloride was effective in mediating the formation of a decanyl glucoside under reasonably mild conditions in good yield, however, the stereochemical preference for the α-anomer was quite poor (pathway b, Scheme 6) [39].

### 3 Indirect activation of the anomeric center for glycosylation reactions

In this section we highlight protecting-group-free strategies that go through what we term an indirect method to activate the anomeric center. In these strategies, the actual glycosylation coupling takes place in the absence of protecting groups. However, the donor applied in the reaction does require the use of protecting groups to access. In each example we first analyze the glycosylation step, but to put the reaction into context we also discuss the synthesis of the donor (if available). Despite this is very major drawback, many of the methods are very innovative and creative and are certainly worth mentioning.

#### 3.1 Remote intramolecular activation

**Scheme 7:**
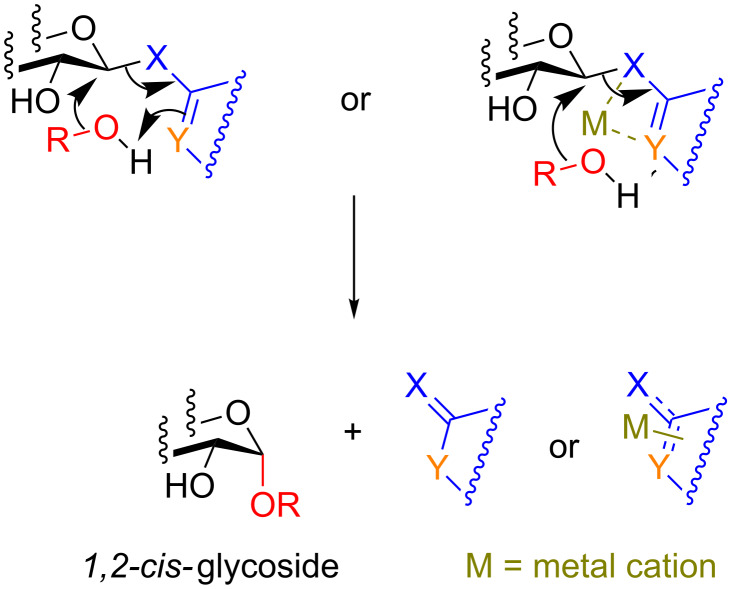
The basis of remote activation (adapted from [37]).

First we focus on the concept of remote intramolecular activation which has been primarily studied and developed by Hanessian and colleagues over the course of 30 years [37]. Because of a thorough review by his group, we discuss his past contributions only before turning to modern approaches that have been built upon his group’s initial work. The foundation of this method is that the activation of the anomeric center of the donor molecule is effected by an interaction between a promotor and an atom not directly attached to the anomeric position (Scheme 7). The anomeric activating group contains two heteroatoms, X and Y that can be activated at the remote atom (Y) by an electrophilic species (H of an alcohol) or a metal cation resulting in a reactive intermediate. This complex could then undergo an S_N_2-like attack of a hydroxy group to furnish the glycoside with inversion of stereochemistry at the anomeric position.

**Scheme 8:**
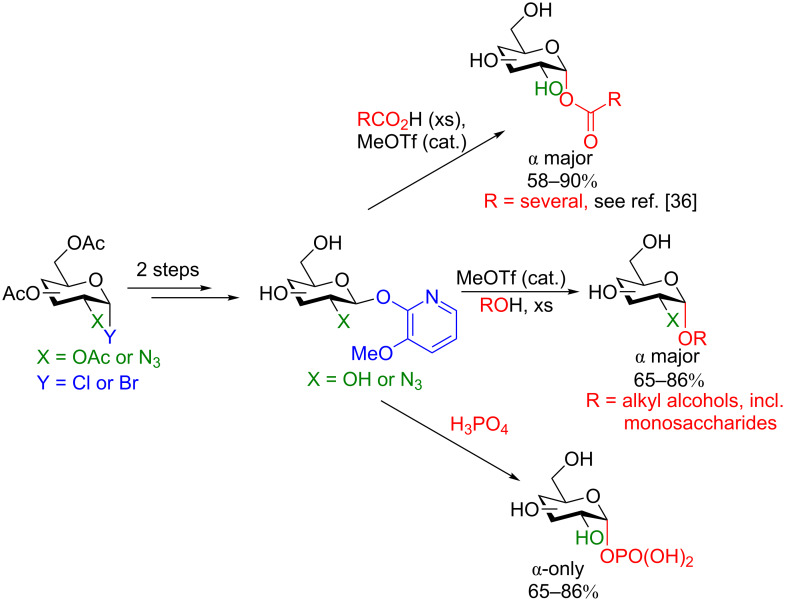
Classic remote activation employing a MOP donor to access α-anomeric alcohols, carboxylates, and phosphates.

**3.1.1 Pyridyl donors for activation:** We first focus on the scope of Hanessian and colleagues’ classical work before we discuss in more detail a very recent example from his lab. The majority of their work employed a 3-methyoxypyridyl (MOP) activating agent at the anomeric position which could provide under the right conditions, regioselectively and stereoselectively 1,2-*cis* glycosides of a wide variety. The substrate scope is broad and even includes glycosyl phosphates and esters in addition to a host of alcohol acceptors (Scheme 8). Certainly the most important application of this chemistry is its ability to provide 1,2-*cis* glycosides with good stereoselectivity, as these glycosides still remain among the most challenging stereoisomers to synthesize as C2 neighboring group participation is not possible [40]. In fact, the access to 1,2-*cis* glycosides is considered a major impetus for the progress in synthetic carbohydrate chemistry [41]. Also noteworthy is the fact that their conditions were applicable to multiple functional groups at C2, as their mechanistic proposal does not include an involvement of this carbon. However, the obvious drawback of this procedure is the extensive protecting group chemistry to synthesize the parent donors (minimum four steps from the unprotected, commercially available saccharides) to access the deprotected MOP-donor for the glycosylation.

**Figure 1:**
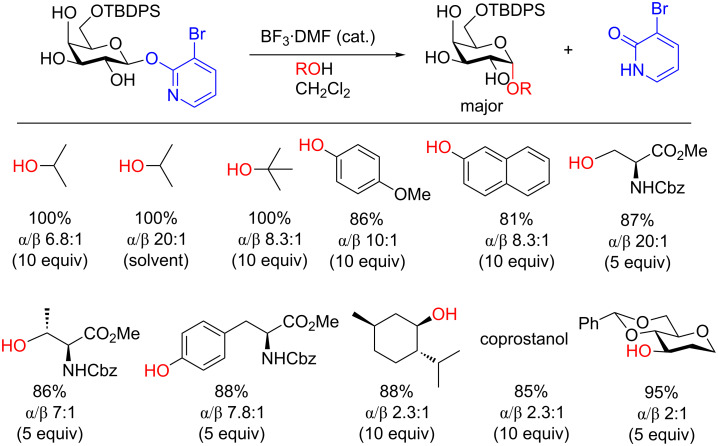
Synthesis of monoprotected glycosides from a (3-bromo-2-pyridyloxy) β-D-glycopyranosyl donor under Lewis acid-catalyzed conditions [42].

In 2016, the Hanessian group revealed a new activating group for the synthesis of monoprotected 1,2-*cis* galactopyranosides in good yield [42]. The conditions were also shown to be feasible for solid-phase synthesis. By employing BF_3_·DMF, the 1,2-*cis*-monoprotected galactopyranosides were obtained in excellent yield and good diastereoselectively in a short time (usually 30 min). The conditions worked well with not only simple aliphatic and phenolic alcohols but also with amino acids and steroid alcohols (Figure 1).

**Scheme 9:**
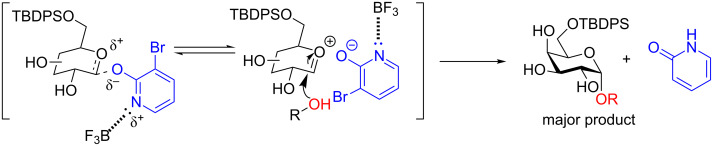
Plausible mechanism for the synthesis of α-galactosides. TBDPS = *tert*-butyldiphenylsilyl.

The authors posit that the stereoselectivity can be rationalized by an oxycarbenium/BF_3_-coordinated 3-bromo-2-pyridyloxy ion-pair intermediate that is displaced by the alcohol in an S_N_2-like reaction (Scheme 9).

**Scheme 10:**
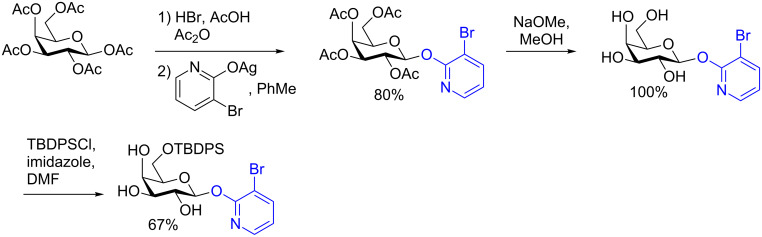
Synthesis of the 6-*O-*monoprotected galactopyranoside donor for remote activation.

The most unfortunate drawbacks of the procedure include the need for a large excess of the alcohol acceptor and the multistep synthesis of the galactosyl donor. The synthesis of the monoprotected activated galactosyl donor requires the use of protecting groups, however, it comprises only three steps from the peracetylated galactose moiety and is high yielding (Scheme 10) [42].

**Scheme 11:**
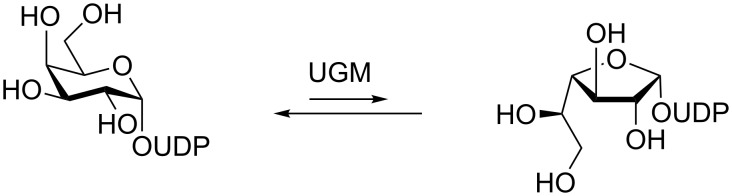
UDP-galactopyranose mutase-catalyzed isomerization of UDP-Gal*p* to UDP-Gal*f.*

**3.1.2 1-Thioimidoyl donors for activation:** Over the last ten years, Plusquellec, Ferrières and co-workers [43] applied the concept of remote activation for the construction of furanosylhexose saccharides. The interest in hexofuranoses is based on the arabinogalactan-rich membrane of *Mycobacterium tuberculosis* and other harmful microorganisms which consists of primarily Ara*f* and Gal*f* subunits [44]. One key step in the biosynthesis of these hexofuranoses is the isomerization of uridine 5′-diphospho (UDP)-pyranose to the corresponding furanosyl donor catalyzed by pyranose mutases. Shown is the transformation of UDP-Gal*p* to UDP-Gal*f* catalyzed by UDP-galactopyranose mutase (UGM) (Scheme 11) [44–45]. A more expedient access to hexofuranose analogs could have implications in the study of infection and potential treatments.

**Scheme 12:**
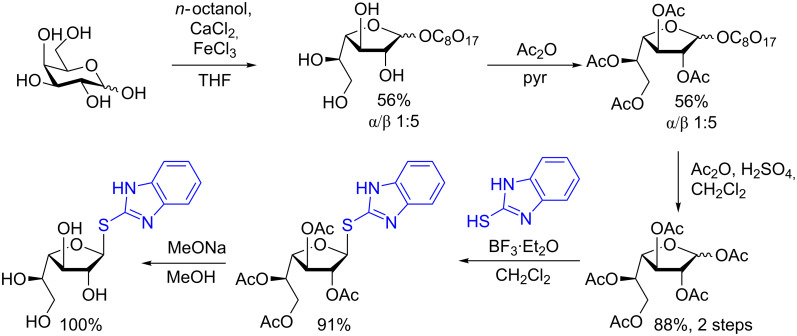
Synthesis of the 1-thioimidoyl galactofuranosyl donor.

Their initial work describes the direct synthesis of 1-*O*-phosphofuranosyl hexoses from the corresponding 1-thioimidoyl donor. The donor is available in good yield over five steps starting from the parent monomer unit using a methodology they previously developed (the synthesis of the Gal*f* donor is shown in Scheme 12) [46–48]. Although certainly lengthy (5 steps from Gal*p*), the 1-thioimidoyl donor is accessible through straightforward chemistry and finds wide application.

The subsequent treatment of the donors with phosphoric acid in DMF at room temperature provided 1-*O*-phosphofuranosyl hexoses in good to excellent yield (Table 1, entries 1–4) [43]. The conditions provided only a very modest stereoselectivity, however, the α anomer was slightly favored regardless of the stereochemistry of the sugar at C2. Most importantly, very little or no ring expansion to the pyranose was observed in any instance.

**Table 1:** Protecting-group-free synthesis of hexofuranosyl 1-phosphates from hexofuranosyl 2-thiobenzimidazole donors using remote activation.

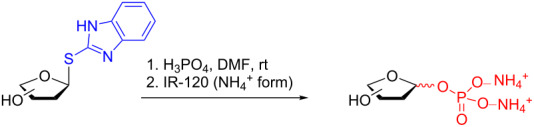				

Entry	Donor moiety	Yield	Glycosylation time (min)	α/β ratio

1	D-Gal*f*	58%	18	1.2:1
2	D-Glc*f*	48%	90	1.2:1
3	D-Man*f*	67%	15	1.6:1
4	D-Fuc*f*	90%	20	1.5:1

In a 2007 follow-up study, Plusquellec and colleagues optimized a Lewis-acid-directed glycosylation approach in the presence of divalent cations to synthesize galactosyl furanosides using the same thioimidoyl-activating group. The optimized conditions (Table 2) allowed for the synthesis of not only simple alcohols (Table 2, entry 1), but also disaccharides, if the accepting hydroxy group is primary (Table 2, entries 2–4). Under the optimized conditions no ring expansion was observed and a very modest preference for the α anomer was detected [49].

**Table 2:** Protecting-group-free *O-*glycosylation using a galactofuranosyl 2-thiobenzimidazole donor.

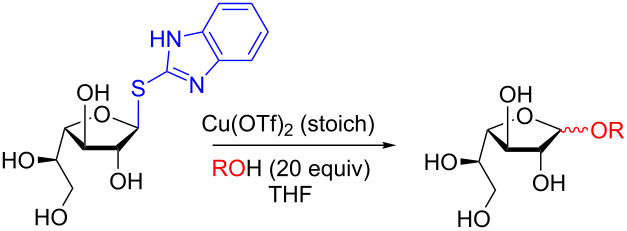				

Entry	Acceptor (R)	Yield	Glycosylation time (h)	α/β ratio

1	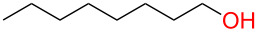	71%	24	1:4.7
2	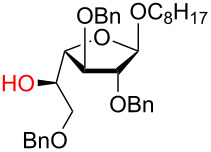	decomposition	–	–
3	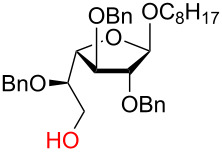	41% 47%	0.5 24	1:1.7 1:3.7
4	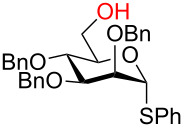	41%^a^	24	1:7.1

^a^Yield after acetylation of the donor -OH groups (Ac_2_O–pyridine).

In their most recent study, the same activating group was employed for the synthesis of UDP-furanoses which were applied in the study and discovery of other UDP-pyranose mutases [50]. Excitingly, their remarkably simple procedure provides the UDP-furanose analogs in moderate yields, however, with the exception of L-Ara*f* (Table 3, entry 2) the reaction is not overly stereoselective for the α anomer with the β anomer sometimes even presenting in excess (Table 3, entry 1).

**Table 3:** Protecting-group-free UDP-furanoses using furanosyl 2-thiobenzimidazole-based donors.

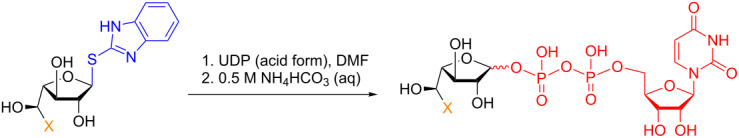					

Entry	X	Yield	Glycosylation time (min)	Temp. (°C)	α/β ratio

1	CH_2_OH (D-Gal*f*)	32%	10	0	1:2
2	H (L-Ara*f*)	31%	8	−10	1:0
3	CH_2_F 6F-D-Gal*f*	37%	60	0	1.3:1
4	CH_3_ D-Fuc*f*	27%	10	0	2:1

This remote activation offers a tremendous potential as viable synthetic option to access otherwise difficult-to-obtain hexose furanosides. These may find application as enzyme substrates and possibly as inhibitors in several bacterial diseases, as well as in solid-phase-oligosaccharide synthesis, as shown. However, the main drawback is still the multistep synthesis of these anomeric activating donors.

#### 3.2 Self-activation of the anomeric center

**Scheme 13:**
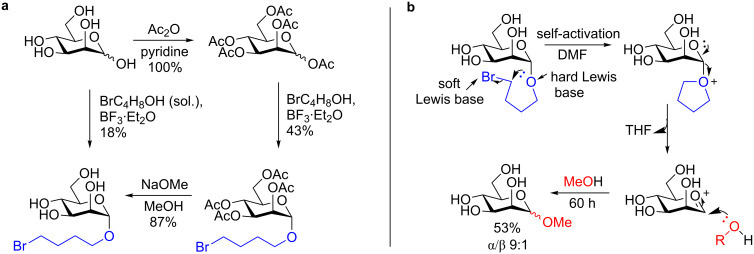
Glycosylation of MeOH using a self-activating donor in the absence of an external activator. a) Synthesis of the 4-bromobutanyl donor. b) Proposed mechanism.

Davis and colleagues [51] developed a unique glycosylation strategy that employs a 4-bromobutanyl group as a self-activating aglycone on a mannose monomer (Scheme 13) which works even in the absence of any activating agent, such as TMSOTf. The synthesis of the self-activating donor proceeds in one (very low yielding) or in four steps from D-mannose in a straightforward manner (Scheme 13a).

In the proposed mechanism (Scheme 13b), the anomeric oxygen self-displaces the bromide (hard Lewis base, soft Lewis base pairing) at the 4-position to form a THF ring. The ring oxygen then displaces THF hence forming an oxocarbenium ion. The latter is subsequent attacked by the nucleophilic alcohol to provide the 1,2-*trans* glycoside with good diastereoselectivity and moderate yield.

This methodology holds tremendous potential if the alcohol acceptor is a saccharide moiety as the diastereoselectivity of the reaction is good, the mechanism unique, and the synthesis of the donor is reasonably easy and inexpensive. However, to date no follow-up study has been published.

#### 3.3 Lewis acid-mediated activation

**Scheme 14:**
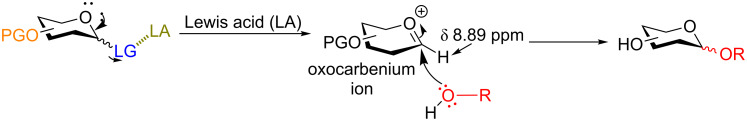
The classical Lewis acid-catalyzed glycosylation.

Lewis acid-mediated methods for anomeric activation of protected donors in glycosylation are very common and well-studied. Typical Lewis acids employed for anomeric activation are TMSOTf and BF_3_·Et_2_O (Scheme 14). The reactions proceed through an oxocarbenium ion that was very recently observed by NMR under cryogenic (−40 °C) conditions stabilized by the HF/SbF_5_ superacid [52]. The highly electrophilic carbon adjacent to the oxocarbenium ion then reacts with the nucleophilic acceptor in either and S_N_1 or S_N_2-like mechanism depending on the chemical stability of the glycosyl cation [53]. The stereochemical outcome of the reaction is generally dictated either by neighboring-group participation of position 2 on the ring or by the anomeric effect when neighboring group participation is not possible [54–55].

Seemingly impossible Lewis acid-mediated processes are then available for the other hydroxy groups on the unprotect donor molecule and some examples are highlighted below.

**Figure 2:**
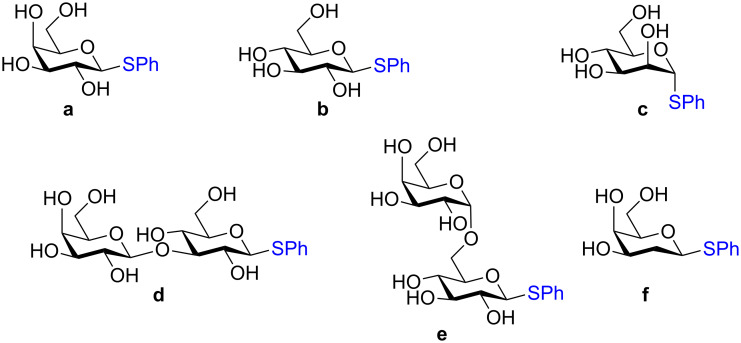
Unprotected glycosyl donors used for the Lewis acid-catalyzed protecting group-free glycosylation reaction to access 1,2-*cis* glycosides.

**3.3.1 Access of 1,2-*cis* glycosides:** A potentially attractive strategy for a 1,2-*cis* glycosylation has been described by Baker and colleagues [56] and employs the use of a deprotected thiol glycoside in the presence of a large excess of Lewis acid (TMSOTf) and *N*-iodosuccinimide (NIS). Although the stereoselectivity of the reaction is noteworthy and the study extensive (Figure 2, Table 4), the authors were unable to isolate the glycoside products without acetylating the free hydroxy groups in the reaction mixture prior to purification. However, one very interesting finding is that their conditions are still modestly α stereoselective when a 2-deoxygalactose analog is used as the donor and propargyl alcohol as the acceptor (Table 4, entry 13).

**Table 4:** 1,2-*cis* Glycosylation of glycosyl thiols.

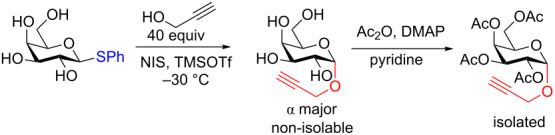				

Entry	Donor	Acceptor	Isolated yield	α/β ratio

1	**a**	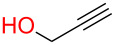	75%	10:1
2	**a**	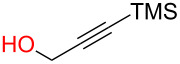	62%	10:1
3	**a**	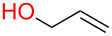	71%	7:1
4	**a**	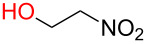	93%	5:1
5	**a**	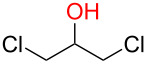	85%	5:1
6	**a**	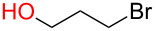	56%	3:1
7	**a**	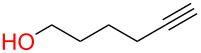	42%	>20:1
8	**a**	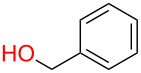	72%	3:1
9	**b**	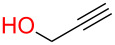	79%	7:1
10	**c**	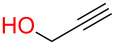	81%	1:2
11	**d**	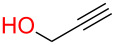	69%	8:1
12	**e**	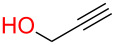	57%	12:1
13	**f**	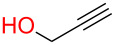	67%	1.7:1

**Scheme 15:**
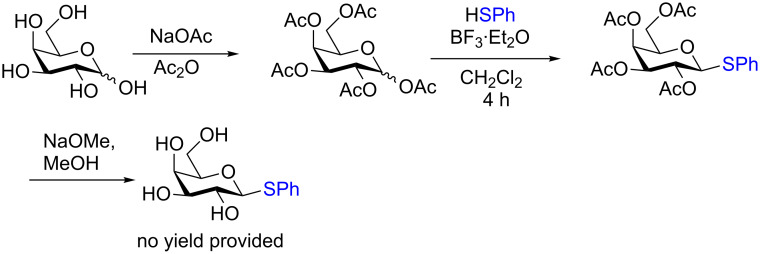
Four-step synthesis of the phenyl β-galactothiopyranosyl donor.

It is crucial to mention that the synthesis of the parent thiol glycoside donors is a multistep process and the authors did not provide yields for the synthesis of any of the glycosyl donors screened in the study. The preparation of the phenyl galactothioside is shown in Scheme 15. The chemistry can be described as straightforward, however, the use of thiophenol is required which is highly toxic and odorous.

**Scheme 16:**
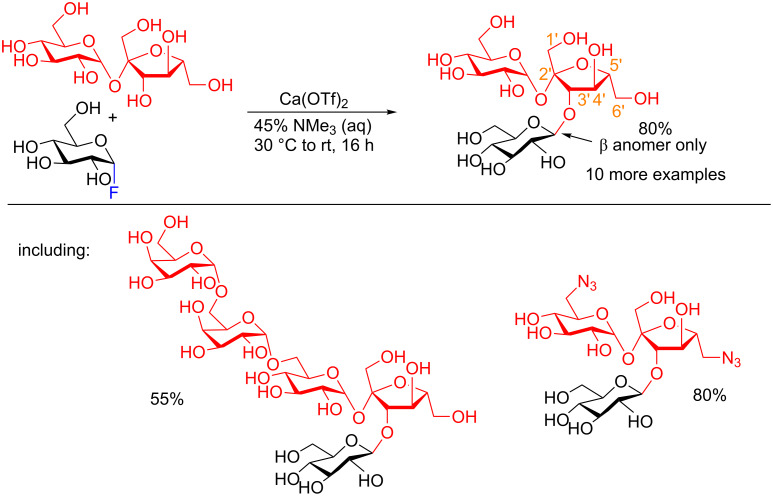
Protecting-group-free C3′-regioselective glycosylation of sucrose with α–F Glc.

**3.3.2 Regioselective glycosylation of unprotected acceptors:** Another remarkably simple protocol that allows for a C3′-regioselective glycosylation of unprotected sucrose under aqueous conditions has been described by Schepartz, Miller and colleagues [57]. They took advantage of the fact that most glycosyl transferase enzymes operate in a divalent metal cation-dependent fashion [58–59]. Therefore, they postulated that by using the correct divalent cation and suitable Lewis acid/Lewis base pairing, the necessary transition-state organization to favor glycosylation of a glycosyl fluoride would outcompete hydrolysis in the aqueous medium. This would lead to a simple non-enzymatic glycosylation procedure. After extensive optimization, the authors obtained the regioselective C3′-glycosylated sucrose analog in very good yield with complete inversion of stereochemistry at the anomeric position (Scheme 16) by using Ca(OTf)_2_ and NMe_3_ (aq).

Two of the most remarkable substrates compatible under these conditions that were still regioselectively glycosylated were stachyose which contains 14 hydroxy groups (Scheme 16, bottom left), all of which could serve as acceptor sites, and the azide analog (Scheme 16, bottom right) which can serve as a potential precursor in the synthesis of aminoglycosides, well-known antibiotics [60]. Even more remarkably, when Ca(OH)_2_ is used in lieu of Ca(OTf)_2_ the 1′-β-glucoside is formed as the major product (ratio of 3′ to 1′ 30:70) in 65% total yield of the two products.

To provide insight into the regioselectivity of the reaction, the authors synthesized sequentially deoxygenated sucrose derivatives and quantified H-to-D isotope exchange effects (from the deuterated solvent) using ^1^H NMR techniques. They concluded that it is the complex hydrogen-bonding network present in sucrose that played the key role in determining the reactivity and selectivity of the reaction. Removal of the OH groups at positions C2 of Glc, C1′and C3′-Fru resulted in no conversion at all. The authors demonstrated that not only these deoxygenations change the hydrogen-bonding network but also have a very large effect on the overall nucleophilicity of the disaccharide and its corresponding interactions with Ca^2+^ under the reaction conditions.

**Scheme 17:**
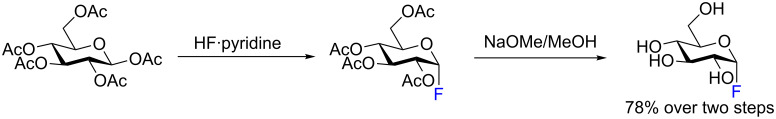
Synthesis of the α-fluoroglucosyl donor.

One drawback of this method is the synthesis of the α-fluoroglucosyl donor which included protecting group manipulations, however, the procedures are reasonably simple and well established (Scheme 17) [57]. In two steps and 78% overall yield from β-D-glucose pentaacetate, α-fluoro-D-glucose can be obtained with the key fluorination effected by using Olah’s reagent.

The appeal of this procedure is obvious. It is an operationally simple and stereo- and regioselective method to obtain tri- and oligosaccharides containing a sucrose moiety. What will certainly be interesting in the future if a more in depth mechanism can be discerned.

#### 3.4 Transition metal-catalyzed glycosylation

The metal-catalyzed activation of the anomeric center has been employed in carbohydrate chemistry for many decades and continues to be a very rapidly expanding field. It is driven by the appeal of waste reduction in chemical synthesis that the use of stoichiometric amounts of reagents, unfortunately cause. Typical transition metals employed for promoting the glycosylation of protected acceptors using protected donors include Pd, Ni, Au, Rh, Ru, and Ti [61]. In this section we highlight some very elegant examples of transition metal-catalyzed glycosylation strategies that have been successful even in the presence of other unprotected hydroxy groups in the molecules.

**Figure 3:**
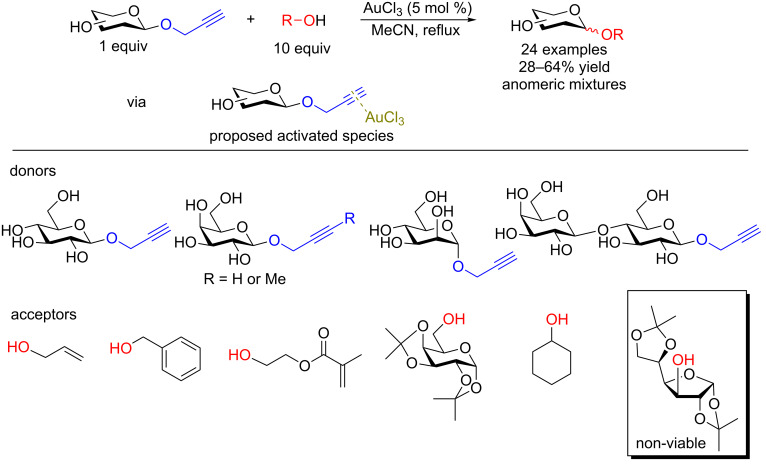
Protecting-group-free glycosyl donors and acceptors used in the Au(III)-catalyzed glycosylation.

**3.4.1 Au(III)–alkynyl complexation:** The Finn group [62] developed a protecting-group-free Au(III)-catalyzed strategy to access both simple aliphatic glycosides and disaccharides in good yields using either a propargyl or 2-butynyl glycosyl donor. They argued that since Au(III) is not too oxophilic and is also working in aprotic solvents, this metal would be suitable for anomeric activation of an alkynyl aglycone. Although used in large excess (10 equiv), all primary alcohols screened in the study (Figure 3) were found to be good acceptors. However, subjecting a secondary alcohol (Figure 3, box) present in diacetone glucose to their conditions lead to cleavage of the 5,6-acetonide only without any glycosylation taking place.

**Scheme 18:**
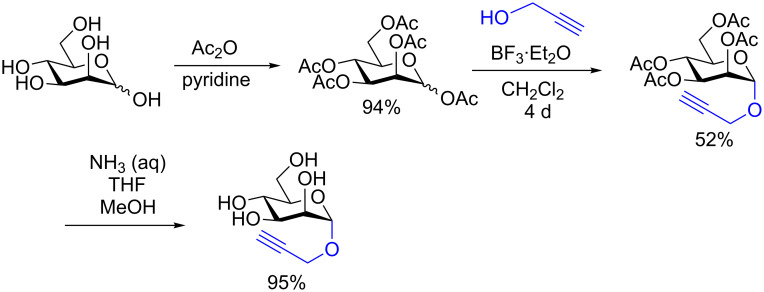
Synthesis of the mannosyl donor used in the study [62].

To our knowledge, this study provides the first example of a metal-catalyzed glycosylation reaction in the presence of free hydroxy groups. The authors postulated that the reaction proceeds through the formation of a π-complex between the alkyne and the metal catalyst [63]. They also determined that MeCN was the most suitable solvent which means that coordination of the ligands to the catalyst is important for progression of the reaction and that if the saccharide donor is acetylated, the reaction does not proceed. It should be emphasized that the reaction suffers from a lack of stereoselectivity and the alcohol acceptor must be used in large excess. It is also unfortunate that multistep syntheses were required to access the donors. However, the synthesis of the donors proceeds through standard, well-described reactions. Shown in Scheme 18 is the synthesis of the mannosyl donor in three steps and 46% overall yield [64].

**Scheme 19:**
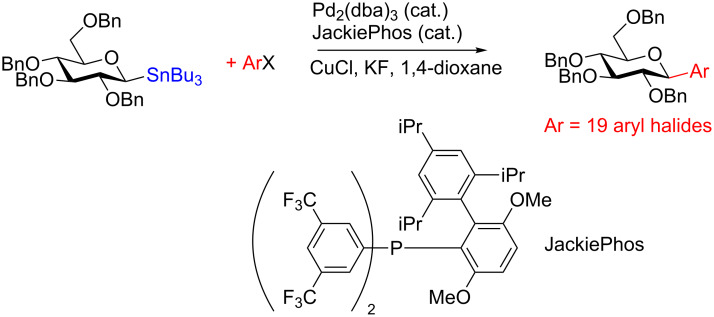
The Pd-catalyzed stereoretentive glycosylation of arenes using anomeric stannane donors.

**3.4.2 Stereospecific Pd-catalyzed glycosylation:** In 2016, Walczak and colleagues [65] described the Pd-catalyzed glycosylation of arenes using anomeric stannanes as donors and aryl halides as the acceptors. The reaction appears to be perfectly stereospecific as complete retention of the stereochemistry was observed in all cases. The initial scope used a benzyl-protected glucosylstannane as the donor and a series of aryl halides as acceptors and in all instances the reaction was stereoretentive (Scheme 19) although a mechanistic rationale remains elusive.

To demonstrate that their conditions were viable in the presence of other hydroxy groups on the saccharides a small series of phenyl *C*-glycosides were synthesized (Table 5). In all cases the reaction was high yielding and perfectly stereospecific for both anomers even in the absence of a C2–OH group (Table 5, entries 5 and 6).

**Table 5:** Stereochemical retentive protecting-group-free *C*-glycosylation.

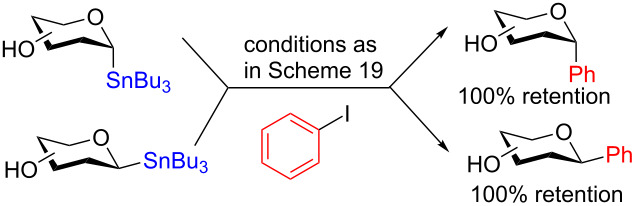		

Entry	Stannanyl donor	Yield of phenyl *C*-glycoside

1	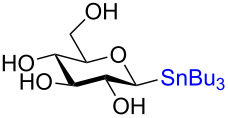	81% (β only)
2	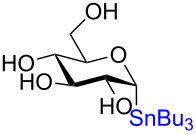	82% (α only)
3	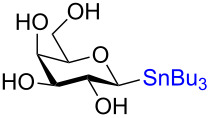	82% (β only)
4	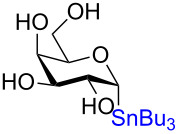	91% (α only)
5	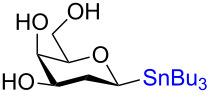	92% (β only)
6	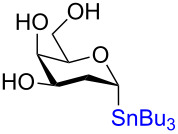	89% (α only)

**Scheme 20:**
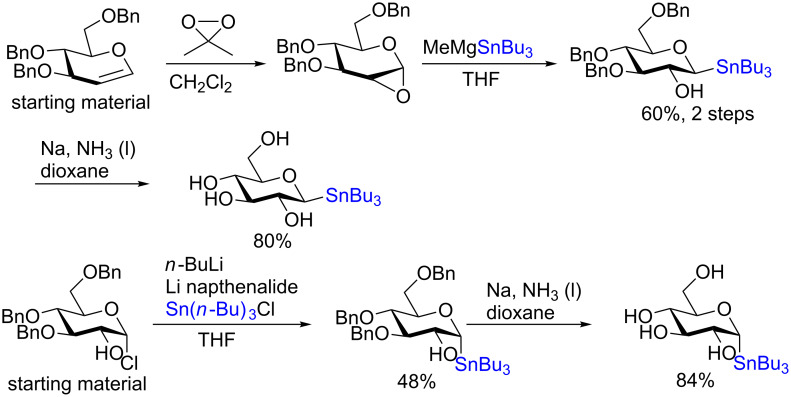
Preparation of the protecting-group-free α and β-stannanes from advanced intermediates for stereochemical retentive *C-*glycosylations.

The remarkable stereospecificity of this reaction coupled with its ability to provide *C*-glycosides, a class of compounds important in natural products [66] and drug design [67], makes this methodology a very powerful one. One very clear drawback, however, is the extremely laborious preparation of the glucosyl donors. More than ten steps are required for their synthesis and the procedures are difficult and include highly toxic components as is highlighted in Scheme 20. The compounds the authors report as the starting materials are already, in fact, multi-step intermediates and the final debenzylation is achieved by using the highly inconvenient Birch reduction.

### 4 Direct activation of the anomeric center

**Figure 4:**
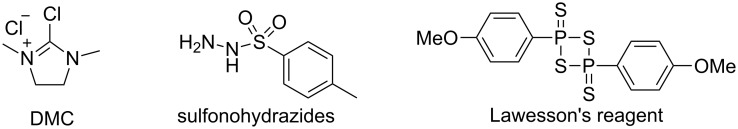
Selective anomeric activating agents providing donors for direct activation of the anomeric carbon.

Over the past 20 years, primarily the Shoda, Fairbanks, and Nitz groups have performed extensive studies on molecules that selectively react at the anomeric position of a saccharide and result in the direct activation at the anomeric carbon. We termed these strategies direct anomeric activation as minimal or no protecting groups to access the parent donors are required. Several methods have been developed and have thus allowed for numerous transformations to occur completely devoid of protection on the other hydroxy groups (Figure 4). Many of these reactions also take place under aqueous conditions which adds increased potential for the widespread use of these agents. These strategies appear more idealized than the indirect activation methods discussed above because the activated donors are available directly from the unprotected saccharide. However, in many instances the scope of the reaction is more constrained so improvements will be welcomed in the future that are almost certainly already underway. In this context the recently published review of Jensen, Thygesen, and co-workers is mentioned as an alternative source as some of the examples from this section also can be found therein [11].

#### 4.1 Activation by 2-chloro-1,3-dimethylimidazolinium chloride (DMC)

Out of the anomeric activating agents that have been developed, by far the widest adopted one is 2-chloro-1,3-dimethylimidazolinium chloride (DMC). In the presence of an excess of DMC and an amine base (typically NEt_3_) mono-, di-, and oligosaccharides are all selectively activated at the anomeric position. Some highlighted examples are described below.

**Scheme 21:**
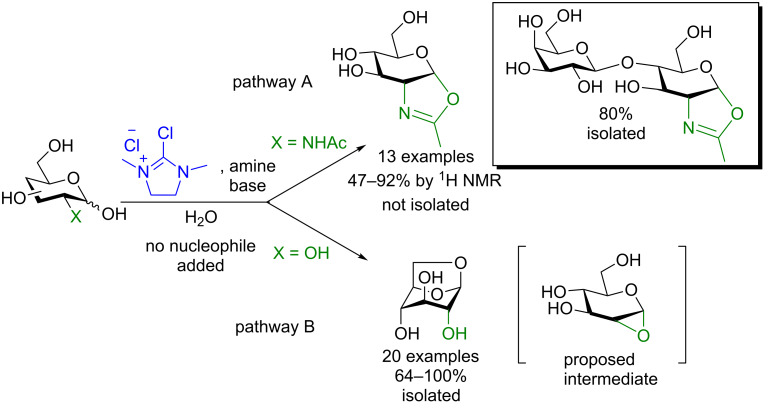
One-step access to sugar oxazolines or 1,6-anhydrosugars.

**4.1.1 Accessing oxazolines and 1,6-anhydrosugars:** The treatment of a 2-deoxy-2-*N*-acetylated sugar with DMC and an amine base in the absence of any nucleophile provides the observable (by ^1^H NMR) or even isolatable (LacNAc) corresponding oxazoline derivative in moderate to very good yield (Scheme 21, pathway A) [68–69]. It is interesting to note that these oxazolines can then be transglycosylated in one pot using a mutant endo-*N*-acetylglucosaminidase [69] (and as reviewed in Noguchi et al. [70]). This demonstrates the tremendous potential of these intermediates not only in classic organic synthesis but also in chemoenzymatic transformations as well.

Under the same conditions but using a C2–OH sugar, the corresponding isolatable 1,6-anhydrosaccharide is formed in good to excellent yield [71]. The authors proposed as the intermediate species the unstable 1,2-anhydrosaccharide resulting from nucleophilic displacement of the activated anomeric center by the C2–OH that is then opened by the primary OH at C6 to form the stable product (pathway B).

**Scheme 22:**
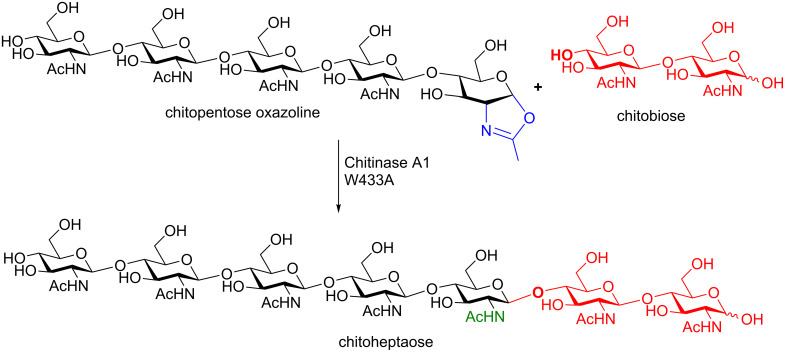
Enzymatic synthesis of a chitoheptaose using a mutant chitinase.

The Shoda group has demonstrated the powerfulness of this one pot access to sugar oxazolines: these oxazolines can be used in transglycosylation reactions using mutant endoglycosidases (Scheme 22). Using a chitinase A1 W433A mutant (i.e., a low hydrolytic activity mutant) in the presence of the chitopentoase oxazoline donor and chitobiose as the acceptor, chitoheptaose can be synthesized without any hydrolysis detected [72]. Further examples of this powerful donor used in enzymatic transglycosylations are available in a review by Nogushi, et al. [70].

**Scheme 23:**
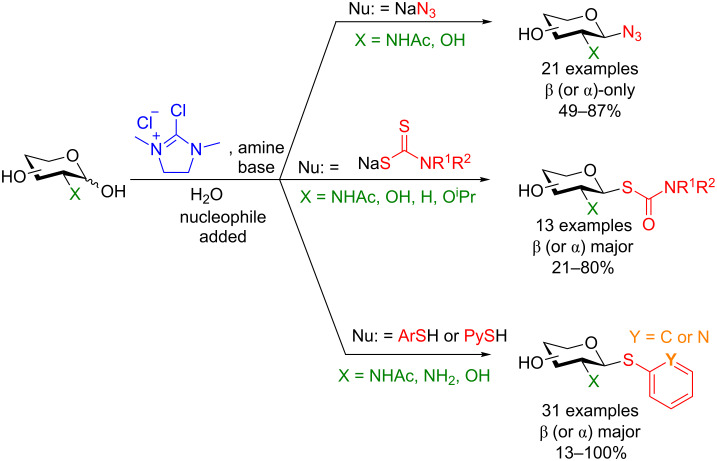
One-pot access to glycosyl azides [73], dithiocarbamates [74], and aryl thiols using DMC activation and subsequent nucleophilic displacement [75–76].

**4.1.2 Synthesis of glycosyl azides, dithiocarbamates, and thiols:** Shoda and colleagues have also shown that when using similar conditions to those described above in the presence of certain nucleophiles glycosyl azides [73], dithiocarbamates [74], and aryl thiols [75–76] can be formed in moderate to quantitative yield with the 1,2-*trans* diastereomer prevailing in all instances (Scheme 23) (also partially reviewed in Noguchi et al. [70]). The substrate scope was extensive in all instances and included successful glycosylation of up to decasaccharide donors.

**Scheme 24:**
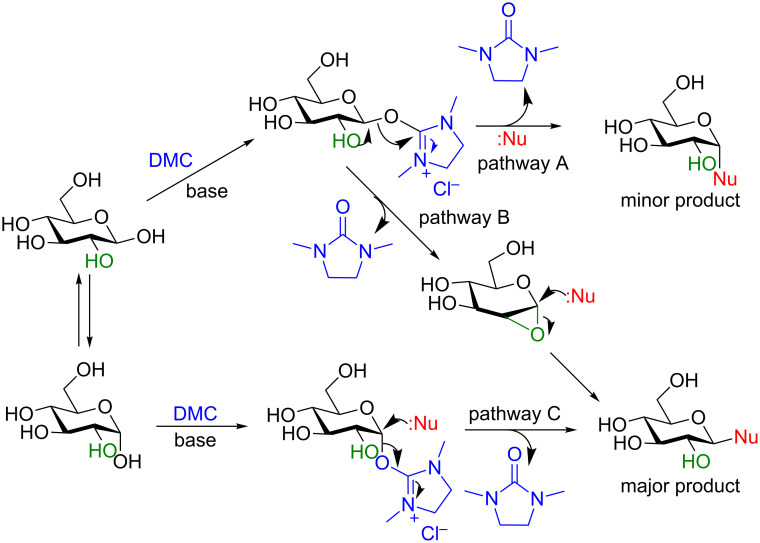
Plausible reaction mechanism.

Shoda and co-workers proposed the differential reactivity at the anomeric center (Scheme 24, glucose shown for convenience) can be explained by the lower p*K*_a_ (≈12.5–14) of the C1–OH [9–10,77]. They believe that the base-promoted nucleophilic attack of the anomeric OH (either the α or β anomer) at the 2-position of DMC results in the formation of the activated anomeric center. This allows for two pathways (B and C) to provide the major (or sole) β-anomer product and only one to form the minor (or unseen) α-anomer product (pathway A). In pathway B, a 1,2-anhydro intermediate is generated by participation of the C2–OH group to release 1,3-dimethylimidazolidin-2-one (DMI), the anomeric leaving group. The epoxide is then opened by the nucleophile when added to the reaction mixture. In pathway C the α-DMC complex is displaced directly by the added nucleophile to liberate DMI, once again to provide the 1,2-*trans* β-diastereomer. Only in pathway A, by direct nucleophilic displacement of the β-activated complex, does the minor α-diastereomer form.

To demonstrate the potential utility of this methodology in labeling strategies, the Shoda group has also accessed fluorescent thioglycosides using the same one-pot strategy [78]. In a follow up work by Novoa et al. [79] by using NEt_3_ and DMC, *S*-linked glycopeptides at cysteine residues on a solid phase could also be obtained. This methodology or very similar variations thereof is now being utilized by several laboratories for various applications and we highlight these examples below.

**Scheme 25:**
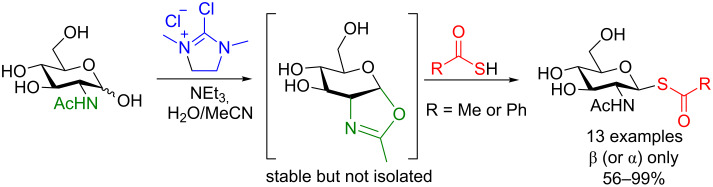
Protecting-group-free synthesis of anomeric thiols from unprotected 2-deoxy-2-*N*-acetyl sugars.

In 2016, the Rademann group could obtain 2-deoxy-2-*N*-acetyl glycosyl thiols using DMC, NEt_3_, and either BzSH or AcSH (Scheme 25) [80]. The authors noticed that the reaction proceeds through the detected oxazoline intermediate to provide solely the β-glycosyl thiol in moderate to excellent yield. They proposed that the oxazoline intermediate blocks access to the α-face of the molecule, hence accounting for the excellent stereoselectivity of the reaction.

**Scheme 26:**
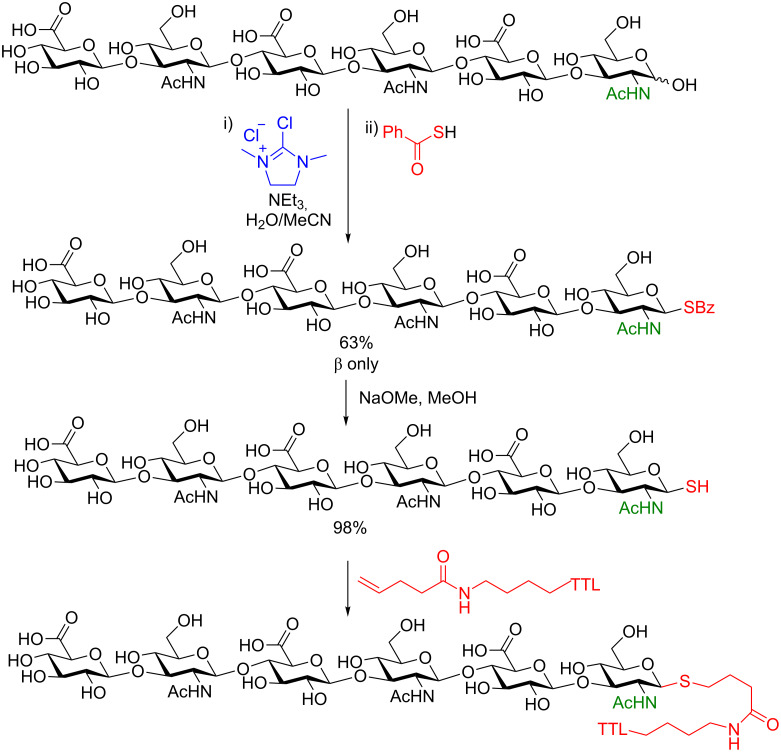
Protein conjugation of TTL221-PentK with a hyaluronan hexasaccharide thiol.

They then demonstrated the power of this method as a ligation strategy. The protected thiol can be debenzoylated or deacetylated using standard conditions (NaOMe/MeOH) nearly quantitatively and then covalently linked to several electrophiles, including proteins. Shown in Scheme 26 is the cross-linking of 1-thiohexahyaluronane (HA-6-SH) to a protein, the engineered thermostable lipase TTL from *Thermoanaerobacter thermohydrosulfuricus*, which contained an unnatural *N*-pentenoyllysine residue at position 221. They incorporated this *N*-pentenoyllysine amino acid into the protein using stop-codon suppression [81–82]. The subsequent thio–ene ligation resulted in quantitative cross-linking to the protein (as determined by ESIMS) [80]. Soon after this seminal work appeared, in 2017, Fairbanks and collaborators constructed a 16-mer peptide with a complex bi-antennary *N*-glycan moiety ligated to the peptide using this thio–ene reaction under very similar conditions [83].

**Scheme 27:**
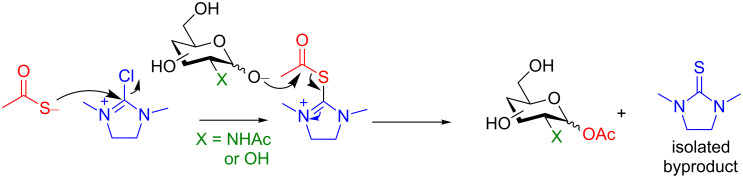
Proposed mechanism.

In 2016, the Fairbanks group devised a one-pot method to obtain glycosyl acetates in the absence of protecting groups [84]. They determined that the order of addition of the reagents was important. First DMC, thioacetic acid and the base are mixed and then the saccharide is added last. Again DMC was utilized as the anomeric activating agent, however, they proposed that first the thiol acetate reacts with DMC to form a thiouronium ester which could be attacked by the deprotonated anomeric alcohol of the saccharide to provide the glycosyl acetate and the isolable thiourea byproduct (Scheme 27).

One key advantage of this strategy is the absence of an amine base in the procedure (Na_2_CO_3_ can be used as the base interchangeably with NEt_3_). These bases are often difficult to remove when purifying polar compounds as the authors mention in their report. When there was an –OH group at C2, the reaction proved to be 100% stereoselective for the 1,2-*trans* product, however, almost a complete lack of stereoselectivity was observed when the C2 contained the NHAc group. We propose that this means, under their conditions, no intermediate oxazoline is formed and therefore there is no neighboring-group participation (Table 6, entries 2, 4, and 6). Encouragingly, disaccharides LacNAc and lactose derivatives are compatible (Table 6, entries 5 and 6) [84].

**Table 6:** Protecting group-free acetylation of unprotected carbohydrates via activation with DMC.

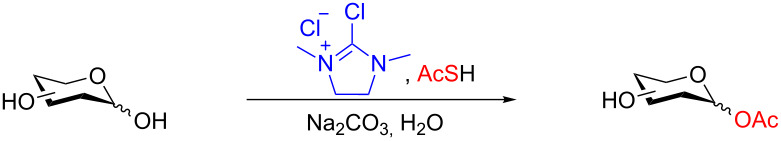				

Entry	Donor	Product	Yield	α/β ratio

1	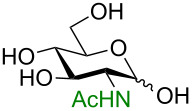	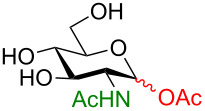	92%	3:2
2	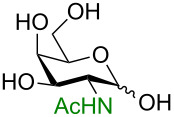	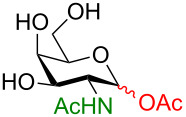	86%	1.1:1
3	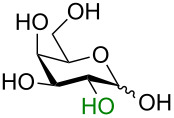	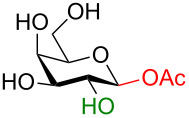	78%	β only
4	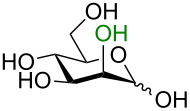	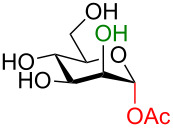	79%	α only
5	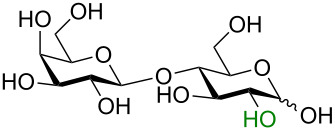	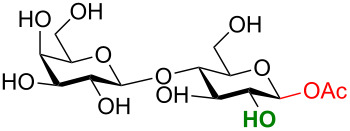	77%	β only
6	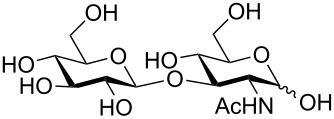	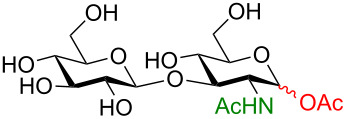	87%	1.1:1

**Scheme 28:**
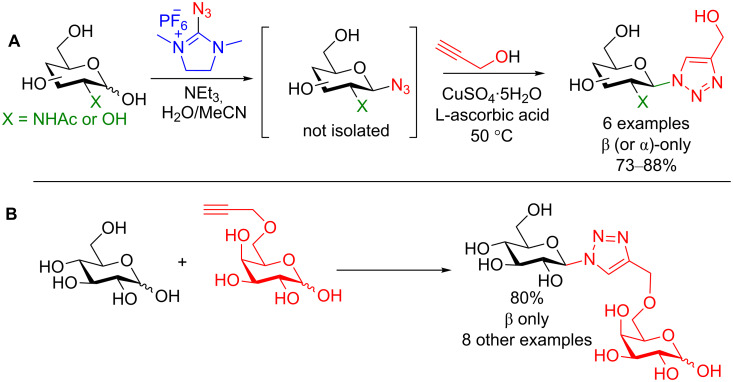
Direct two-step one-pot access to glycoconjugates through the in situ formation of the glycosyl azide followed by the click reaction.

Fairbanks and colleagues have also devised a two-step one-pot method to obtain glycosyl click products [85]. The authors utilized 2-azido-1,3-dimethylimidazolium hexafluorophosphate (ADMI) [86], which very conveniently provides the anomeric activating agent and the nucleophilic azide source on the same molecule (Scheme 28). Under the optimized conditions both 2-deoxy-2-NHAc and C2–OH derivatives could be smoothly transformed to the intermediate azide species. Subsequently by adding an alkyne, CuSO_4_, L-ascorbic acid and applying mild heat, the desired 1,2-*trans* click triazole could be obtained in good to excellent yield [85].

This reaction was shown to be very robust as not only di- and oligosaccharides can be formed (one example is shown in Scheme 28B), but also cancer-associated MUC1 glycopeptides (the alkyne source was propargylglycine in the peptide sequence) which have potential in synthetic vaccines [87–88] in moderate yields (30–47%) [85]. Once again these conditions take place under mild wet conditions, further underscoring the potential of this method.

**Scheme 29:**
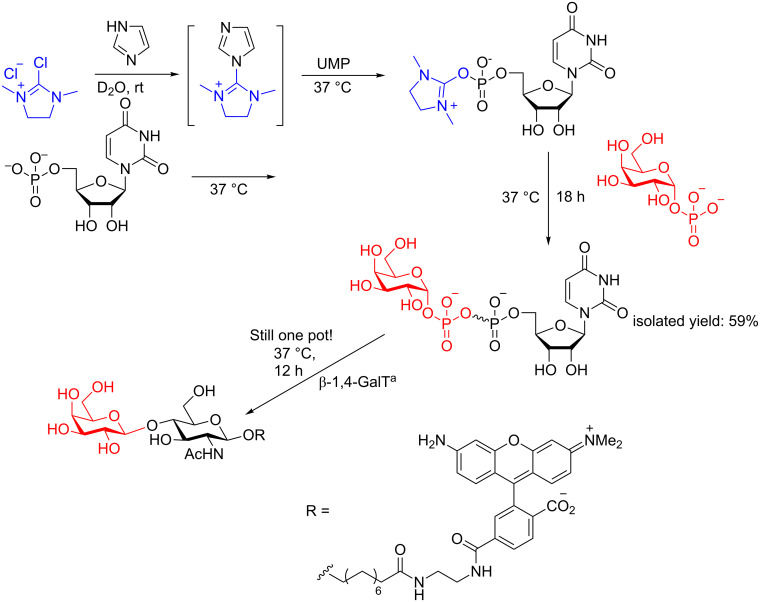
DMC as a phosphate-activating moiety for the synthesis of diphosphates. ^a^β-1,4-galactose transferase.

Due to the mild nature of DMC and its increasing popularity, we briefly highlight the power of this reagent not only in the capacity of glycosylation as an anomeric activator, but also as a phosphate activator to form diphosphates. We selected one example of their work because it also showcases the use of glycosyl transferases in modern protecting-group-free synthesis (Scheme 29) [89]. The Hindsgaul group reacted imidazole and DMC to form an activated species termed 2-imidazolyl-1,3-dimethylimidazolinium chloride (ImIm) which reacted with UMP within one hour to form an activated phosphoester. Then, over 18 h at 37 °C, Gal-1-P can be coupled to form a diphosphate (plus some dimerized UMP–UMP) that is fully isolatable. Adding to the power of this method is the ability for the in situ formed diphosphate to react enzymatically with for example the known GlcNAc analog shown in Scheme 29 using either an inverting (shown) or retaining glycosyl (not shown) transferase in the reaction mixture to form (for example) a LacNAc analog. The power of this methodology is clear: it is a straightforward method to complex oligosaccharides under mild and easy to handle conditions.

#### 4.2 Activation by triazinylmorpholinium salts

**Figure 5:**
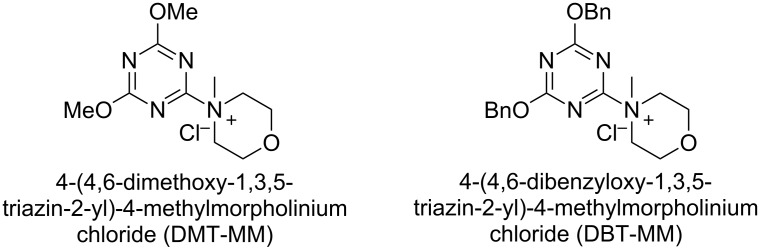
Triazinylmorpholinium salts as selective anomeric activating agents.

In recent years the Shoda group has developed two similar selective anomeric activating agents [90–91] as donors (see previous section) to allow for a two-step metal-catalyzed glycosylation of simple alcohols (Figure 5). In the first step, the anomeric activating agents are generated in situ in aqueous MeCN through the reaction with the corresponding triazine chloride and *N-*methylmorpholine. These agents are then able to selectively react with the anomeric center of a fully unprotected saccharide in the presence of an amine base in a dehydrative manner analogous to DMC from the previous section. These triazine donors are then isolated and reacted in the following step. The power of these two-step strategies is the ability to obtain the much more challenging 1,2-*cis* glycoside.

**Scheme 30:**
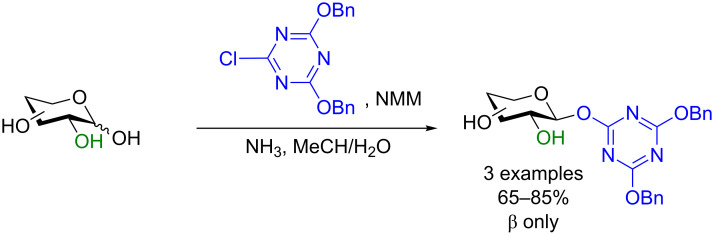
One-step synthesis of DBT glycosides from unprotected sugars in aqueous medium.

In their first study [90] DBT-MM was generated in situ and reacted with three saccharides to form the triazine donor in good yield and perfect stereoselectivity (Scheme 30).

Glycosylation of the resulting DBT-β-glycosides was carried out in various alcohols (selected examples shown in Table 7) under Pd/C-catalyzed hydrogenolysis conditions to liberate the desired 1,2-*cis* glycoside and cyanuric acid. The reductant was either H_2_ (non-alkynyl acceptors) or Et_3_SiH (alkynyl acceptors). In all cases the yields were very good and the stereoselectivity pretty good for the 1,2-*cis* glycoside diastereomer.

**Table 7:** Protecting group-free glycosylation by using DBT donors under hydrogenolytic conditions.

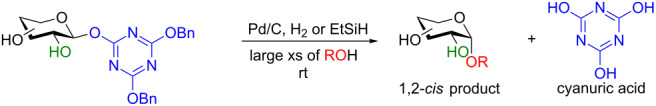				

Donor	Reductant	Acceptor	Yield	α/β ratio

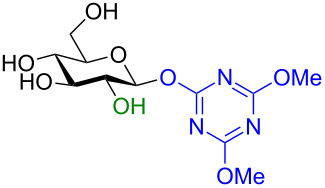	H_2_	MeOH	95%	96:4
	Et_3_SiH	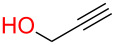	92%	82:18
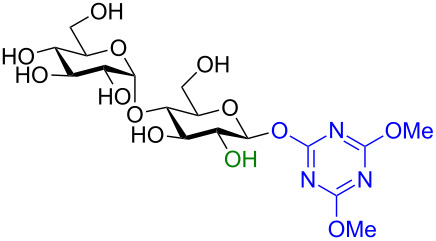	H_2_	MeOH	78%	95:5
	Et_3_SiH	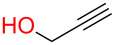	77%	64:36
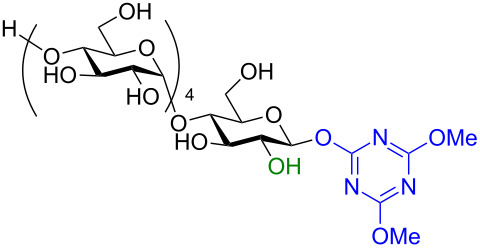	H_2_	MeOH	70%	96:4
	Et_3_SiH	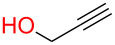	71%	57:43

**Scheme 31:**
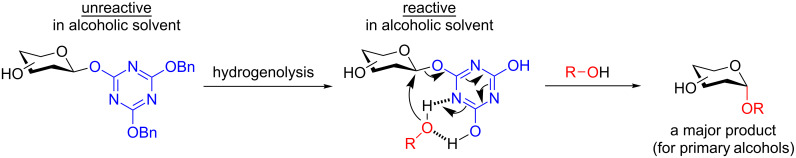
Postulated mechanism for the stereoselective formation of α-glycosides.

The mechanism is thought to proceed through an initial hydrogenolysis of the benzyl groups to produce a reactive dihydroxytriazinyl compound. A subsequent nitrogen activation of the triazinyl ring of this reactive species is proposed to be due to the intermolecular hydrogen bonding with the alcoholic solvent. The alcohol is then delivered in a stereospecific manner to the α-face of the saccharide in an S_N_2-like mechanism for primary alcohols (Scheme 31).

**Scheme 32:**
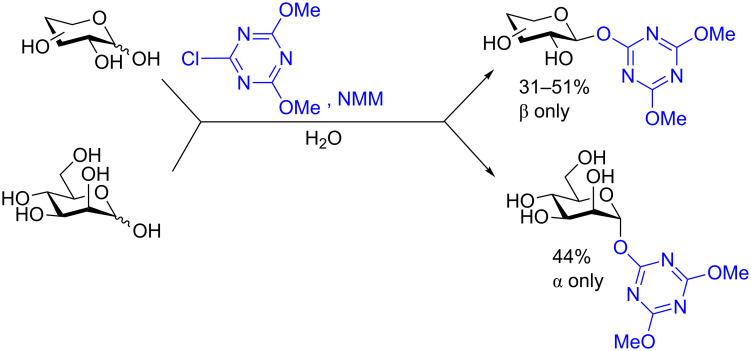
DMT-donor synthesis used for metal-catalyzed glycosylation of simple alcohols.

In a second two-step process [91], the Shoda group designed another catalytic system that provided 1,2-*cis* glycosides in very high yield and often perfect stereoselectivity. In the first step of the reaction the anomeric position is activated by using a dehydrative condensing agent 4-(4,6-dimethoxy-1,3,5-triazin-2-yl)-4-morpholinium chloride (DMT-MM). The isolated yields were moderate but the reaction was perfectly stereoselective for the 1,2-*trans* products (Scheme 32). The product was also formed when using water as the solvent. The DMT glycoside was subsequently treated with 10 mol % of either a Cu(I), Ag(I), or Pd(II) catalyst in the presence of MeOH (or other simple alcohols, not shown) at room temperature. In all cases the yield was either very high or quantitative, the conditions were mild, and the reaction stereoselective for the 1,2-*cis* methyl glycoside (Table 8). The utility of these two methods is obvious as improved syntheses for 1,2-*cis* glycosides are always highly sought and the anomeric activating procedure is simple and can be carried out in water. However, at present both studies were limited to only simple aliphatic alcohols because the glycosylated alcohol was used as a partial solvent. We are certainly interested to see if these methods can be extended to disaccharides or other higher alcohols in the future.

**Table 8:** Protecting group-free metal-catalyzed glycosylation using DMT-based donors to form methyl glycosides with stereochemical inversion.

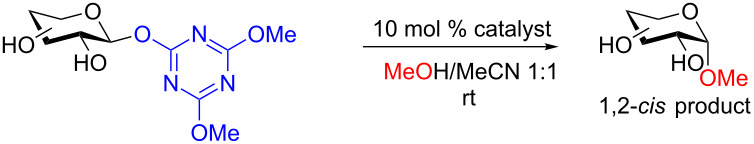			

Donor	Metal(s)	Yield	α/β ratio

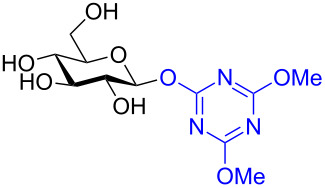	Cu(I), Ag(I), or Pd(II)	All 100%	α-only
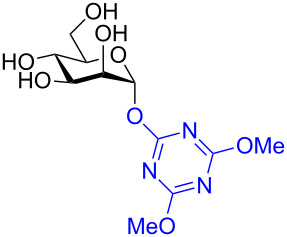	Cu(I), Ag(I), or Pd(II)	84–90%	3:7
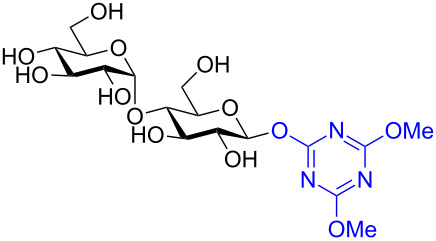	Cu(I)	100%	α-only
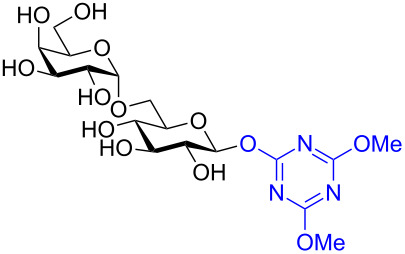	Cu(I)	100%	α-only
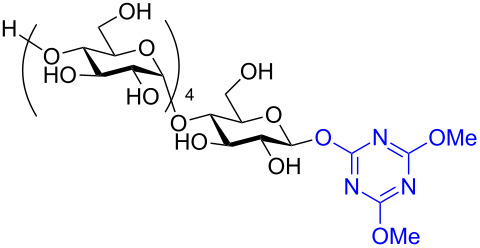	Cu(I)	100%	α-only

#### 4.3 Activation by glycosyl sulfonohydrazines (GSH)

**Figure 6:**
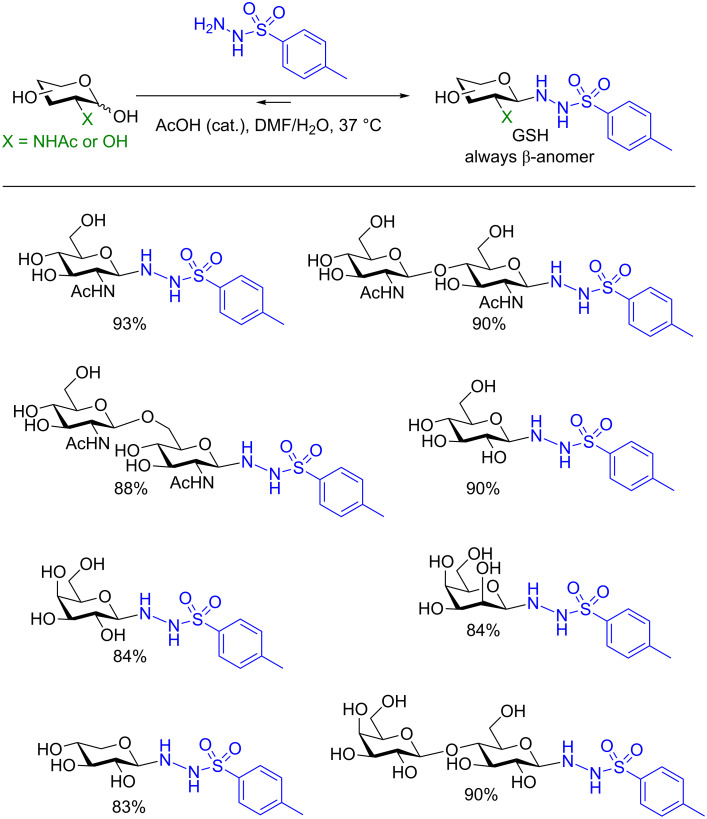
Protecting group-free synthesis of glycosyl sulfonohydrazides (GSH).

In a series of three studies by the Nitz group a sulfonohydrazine activating group was utilized that also reacts selectively at the anomeric position in a condensation reaction. However, the activation takes place in equilibrium under catalytic acidic conditions. On the other hand in the presence of an excess of *p*-toluoylsulfonohydrazide or using concentrated conditions, the authors were able to isolate several exclusively β-glycosyl sulfonohydrazide (GSH) donors in very high yields (Figure 6) [92–93]. Furthermore, both C2–OH and 2-deoxy-2-NHAc saccharides were compatible with their conditions.

**Figure 7:**
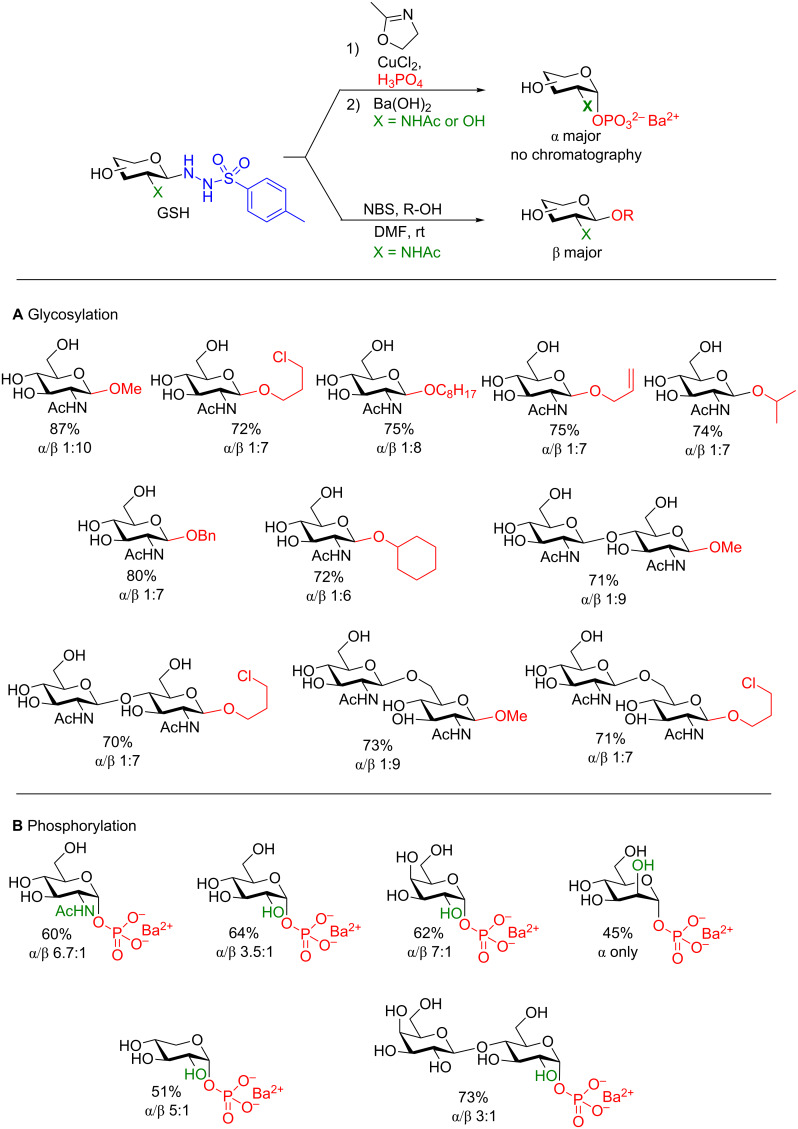
The use of GSHs to access 1-*O*-phosphoryl and alkyl glycosides. **A**) Glycosylation of aliphatic alcohols. **B**) GSHs to access α-glycosyl 1-phosphates.

**Scheme 33:**
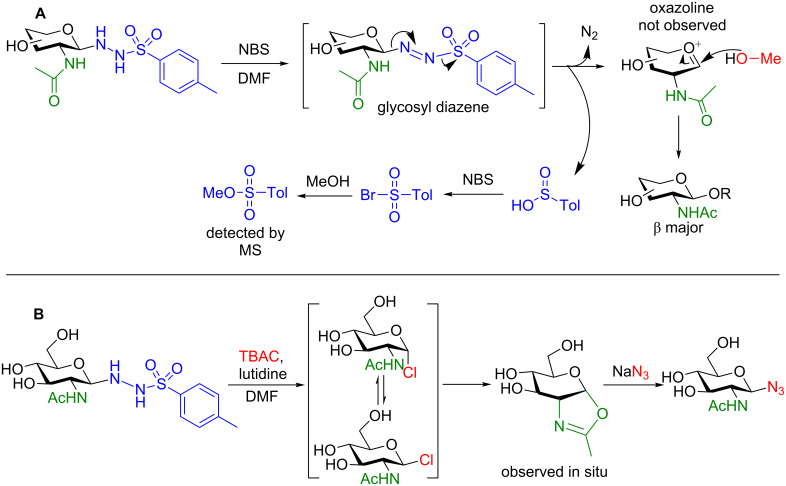
A) Proposed mechanism of glycosylation. B) Proposed mechanism for stereoselective azidation of the GSH donor.

Their initial study [92] focused on the diastereoselective glycosylation of 2-deoxy-2-NHAc sugars using simple alcohols. A sizeable series of glycosides from both mono- and disaccharide donors was accessible in good yield and good β (i.e., 1,2-*trans*)-selectivity (Figure 7A). They proposed a general reaction mechanism, that proceeds through a glycosyl diazene intermediate that is known to form during the oxidation of *N′*-alkylsulfonohydrazides [94] (by NBS in this case) (Scheme 33). The glycosyl diazene then spontaneously collapses to liberate N_2_(g) and sulfinic acid and could provide an oxocarbenium ion to be trapped by either the 2-acetamido group to form an oxazoline (did not occur) or the alcohol (solely observed) in the reaction mixture. The sulfinic acid is further oxidized by NBS to the bromide that could then be displaced by MeOH to provide a stable byproduct. Although the authors reported no incompatible alcohols in their study, 20 equivalents of the acceptor were required to obtain the yields shown in Figure 7A. With such an excess required, this method is still impractical for the synthesis of saccharide–saccharide bonds.

One very noteworthy observation in this study is the ability of these donors to accept a nucleophilic azide source (Scheme 33B). The authors showed that their conditions were feasible for the synthesis of glycosyl azides which are highly useful precursors for *N*-linked glycans [95] or Cu-catalyzed cycloadditions with alkynes (as shown above) [85]. By using tetrabutylammonium chloride and an amine base the highly reactive chlorinated intermediate forms that can be displaced by the 2-acetamido group to provide the observable oxazoline prior to nucleophilic displacement by the azide, N_3_^−^. Due to neighboring group participation, only the β-anomer is observed.

In a 2014 follow-up study using C2–OH GSH donors, the Nitz group reported that, in fact, the anomeric ratio (α:β) depended heavily on the solvent used for the glycosylation. They determined that by selecting the appropriate solvent based on the anomer desired that the ratios could be tweaked [96]. If more work is invested in this area access to 1,2-*cis* glycosides under essentially neutral conditions may be possible.

One very powerful application of this research is the formation of α-glycosyl 1-phosphates under mild conditions with high selectivity [93]. By using simply CuCl_2_ as an oxidant and 2-methyl-2-oxazoline as an additive in the presence of phosphoric acid, α-glycosyl 1-phosphates could be isolated in good yield as the Ba^2+^ salt without the need of any chromatographic purification. The conditions proved to be quite versatile as even a lactosyl phosphate and 1-phospho-α-mannose could be formed as well (Figure 7B). Unfortunately, the authors could not provide any rationale for the stereoselectivity of the reaction.

**Scheme 34:**
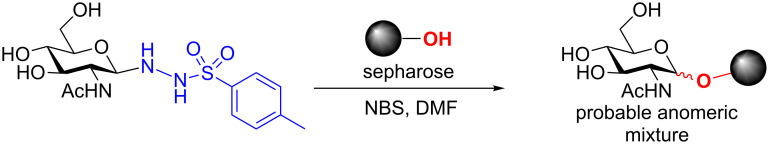
Mounting GlcNAc onto a sepharose solid support through a GSH donor.

Encouragingly this methodology has now being picked up by other research groups. In 2015, Cairo and colleagues used this methodology to mount GSH Gal, Lac, and GlcNAc moieties onto sepharose-binding beads very simply for the use in affinity chromatography which were suitable for binding lectins [97]. GlcNAc binding is shown as an example in Scheme 34.

#### 4.4 Lawesson’s Reagent to form anomeric thiols

**Scheme 35:**
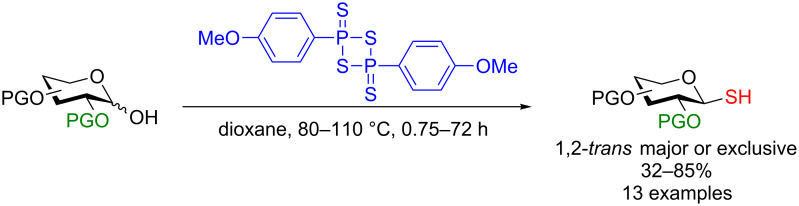
Lawesson’s reagent for the formation of 1,2-*trans* glycosides.

Davis and co-workers described the use of Lawesson’s reagent for the synthesis of both protected and unprotected glycosyl thiols. The study demonstrated tremendous robustness in the synthesis of 1,2-*trans*-protected thiols in good to perfect stereoselectivity (Scheme 35). Furthermore, most protecting groups were compatible and the purification was simple [98].

**Scheme 36:**
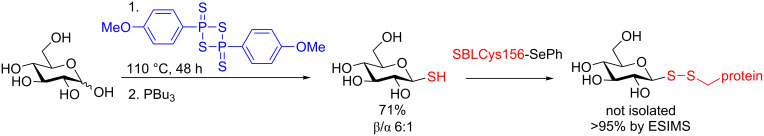
Protecting-group-free protein conjugation via an in situ*-*formed thiol glycoside [98].

The authors reported that the use of unprotected sugars initially proved to be slightly more difficult, due to the tendency of the products to form disulfides. However, it was found that treatment of the crude reaction mixture with PBu_3_ reduced the disulfides allowing for smoother isolation. Importantly, in a second step the unprotected thiol was able to ligate a selenylsulfide-activated single-cysteine mutant protein (subtilisin *Bacillus lentus*, SBLS156C [99]) quantitatively (according to ESIMS) to allow fully protecting-group-free access to glycoproteins. In Scheme 36, glucose is used as an example of this methodology (other sugars in the text) [98].

The potential of this method is clear: a protecting-group-free access to glycoproteins would be a tremendous advancement in the field. The major drawback, however, is that the authors did not isolate the purified glycoprotein; their yield is based solely on the ESIMS analysis of the crude reaction mixture. It is also known that Lawesson’s reagent is not compatible with a C2 NAc group as thionation could also be effected at the amide [100], so somewhat limiting the saccharide scope of this chemistry. We feel that a follow-up study providing the protecting-group-free access to the purified protein would be of great importance.

#### 4.5 pH-Specific activation

**Scheme 37:**
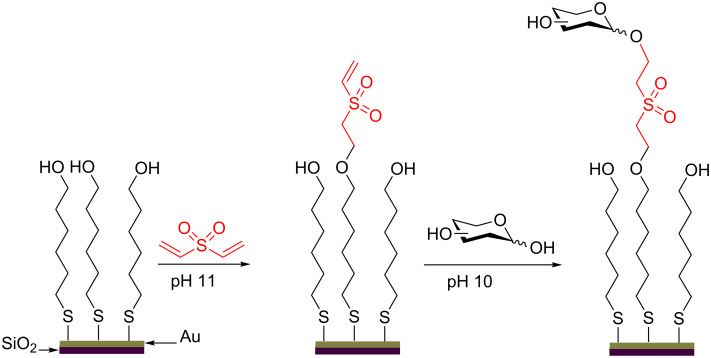
pH-Specific glycosylation to functionalize SAMs on gold.

Protecting-group-free glycosylations have now even been described in materials chemistry. Here they provide an extremely efficient way to functionalize hydroxy-terminated self-assembled monolayers (SAM) on gold (Scheme 37). First the surface was incubated with divinyl sulfone (DVS) in a basic aqueous buffer (pH 11) followed by incubation with the sugar in aqueous buffer at pH 10. In all cases the Michael addition took place regioselectively at the anomeric position of the carbohydrate moiety. Each of the two surface-modification steps as well as the incubation steps was characterized using X-ray photoelectron spectroscopy (XPS) to determine the organic composition [101].

The easiness of this procedure is noteworthy. By simple pH adjustments in a two-step process a functionalized glycan-surface array can be obtained using a fully unprotected saccharide donor under aqueous conditions.

### 5 In situ activation of the anomeric center

In this section we discuss the developments in selective in situ-activation of the anomeric center using reagents that react in a non-pH-dependent manner. Fascinatingly both stoichiometric and catalytic reagents are now available to effect the glycosylation using unprotected saccharide donors.

#### 5.1 Activation using Mitsunobu reagents

Over the last 50 years the Mitsunobu reaction [102] has developed into one of the mainstays in the organic chemist’s toolbox. It has such far reaching potential that it or partial variants of the procedure can now be utilized in glycosylation reactions of unprotected and unactivated carbohydrates to form glycoconjugates. Furthermore, this reaction operates under neutral conditions further increasing its power and potential for widespread application in protecting-group-free glycosylations. However, there are mainly two obvious drawbacks of the operation: First, the formation of difficult-to-remove (especially in the presence of polar, unprotected products) phosphine oxide and second the need to work under anhydrous conditions. However, using stoichiometric conditions the list of glycosides available is now expansive ranging from simple phenolic glycosides to aromatic esters to fully unprotected nucleosides. We highlight these examples below. Due to the inherent challenges in both selective reactivity and purification of such polar products in the presence of phosphine oxide, it is not surprising that extensive optimization of conditions was necessary in all cases.

**Figure 8:**
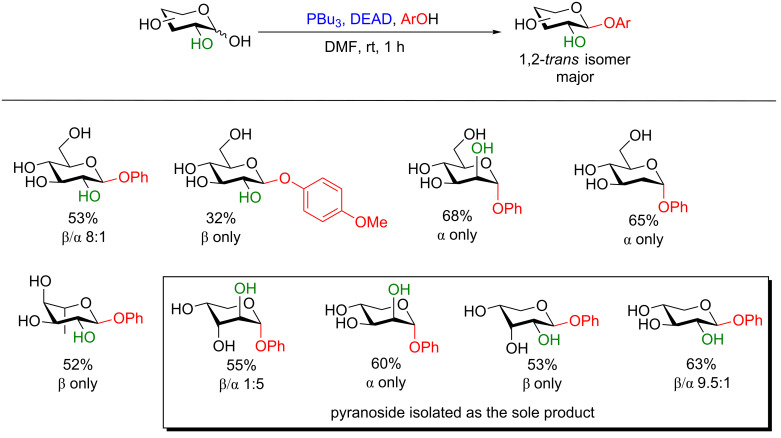
Protecting-group-free availability of phenolic glycosides under Mitsunobu conditions. DEAD = diethyl azodicarboxylate.

**5.1.1 Accessing phenolic glycosides:** To the best of our knowledge the first example of this methodology was applied to the synthesis of phenolic glycosides by Grynkiewicz [103–104] nearly 40 years ago. In his seminal work he discovered that by using a more reactive phosphine (PBu_3_), aryl glycosides could be formed wholly regioselectively for the anomeric position and with good or perfect stereoselectivity for the 1,2-*trans* diastereomer. Also, in the case of furanoses, the thermodynamic pyranoside regioisomer formed exclusively (Figure 8) [104]. Very interestingly, though, in the case of 2-deoxyglucose exclusively the α-anomeric product was observed, but, unfortunately, no explanation for this anomaly has been provided.

Although the author did not provide any definitive mechanistic insight, he suggested that the C2–OH may act as a directing group or is involved in neighboring group participation. This method likely represents the first ever described neutral glycosylation strategy using unprotected and unactivated donors. Although the implications of this methodology are clear, we believe that it has been largely overseen for over 30 years.

**Scheme 38:**
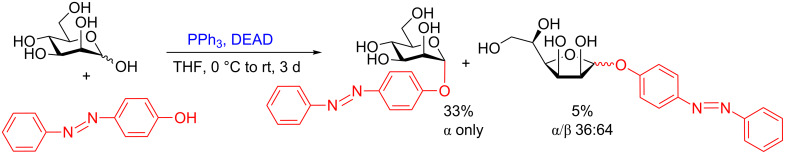
Accessing hydroxyazobenzenes under Mitsunobu conditions for the study of photoswitchable labels. DEAD = diethyl azodicarboxylate.

In 2015 a very similar procedure has been employed for the synthesis of α-mannopyranosyl hydroxyazobenzenes that Lindhorst and colleagues applied to their study of photoswitchable labels. Their procedure furnished the desired aryl α-mannopyranoside in moderate yield with slight contamination by an anomeric mixture of mannofuranosides (Scheme 38) [105].

**Scheme 39:**
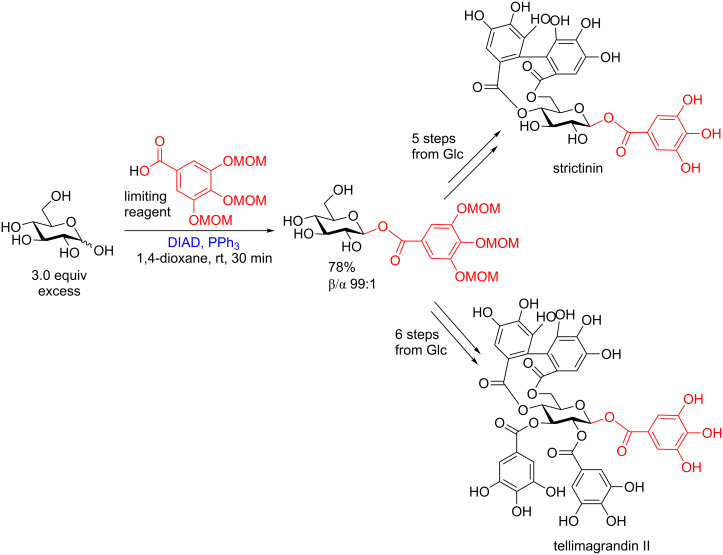
Stereoselective protecting-group-free glycosylation of D-glucose to provide the β-glucosyl benzoic acid en route to the protecting-group-free total synthesis of two ellagitannins. DIAD = diisopropyl azodicarboxylate.

**5.1.2 Esterification of benzoic acids with glycosyl donors:** Again in 2015 Kawabata and co-workers applied the Mitsunobu reaction to an unprotected and unactivated donor in the first step of the total synthesis of two ellagitannins. Using a moderate excess of unprotected D-glucose as the donor and a functionalized benzoic acid as the acceptor, the authors were able to isolate the β-glucosyl benzoic acid in good yield and high stereoselectivity after extensive optimization (Scheme 39). With this benzoic acid in hand the authors succeeded in the synthesis of both strictinin and telligrandin II in 5 steps and 6 steps from D-glucose, respectively, in the absence of protecting groups on the carbohydrate scaffold [106].

**Figure 9:**
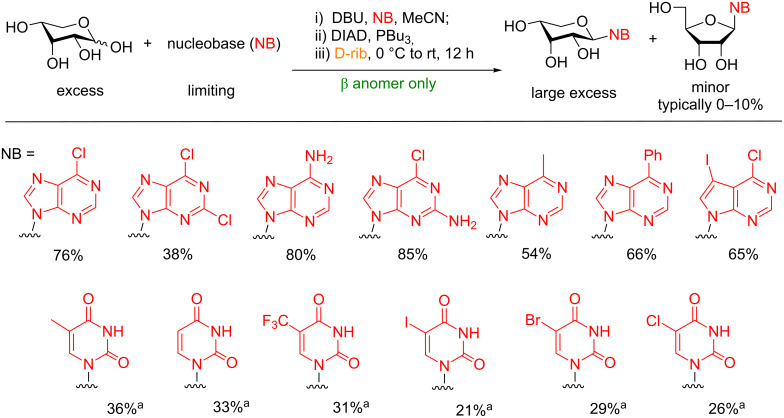
Direct synthesis of pyranosyl nucleosides from unactivated and unprotected ribose using optimized Mitsunobu conditions. ^a^As determined by ^1^H NMR. The products were inseparable from the furanoside using silica gel chromatography. DIAD = diisopropyl azodicarboxylate.

**5.1.3 Synthesis of nucleosides:** Inspired by the previous efforts of Grynkiewicz, we explored the feasibility of synthesizing nucleosides using an optimized Mitsunobu protocol [107]. Extensive optimization of the reaction conditions revealed that the equivalency of the sugar, reactivity of the phosphine, and order of addition of reagents were the most important factors in obtaining satisfactory yields. Under our conditions, unactivated and unprotected ribose could be used to glycosylate both purine and pyrimidine (Figure 9) nucleobases to provide solely β-ribosyl nucleosides in moderate to good yields with the thermodynamic pyranoside regioisomer dominating in all instances over the biologically more relevant furanoside. In general, purine bases proved to be more reactive than the pyrimidines and could be separated from the minor furanosyl byproducts, which was not possible in case of the pyrimidinyl nucleosides.

**Figure 10:**
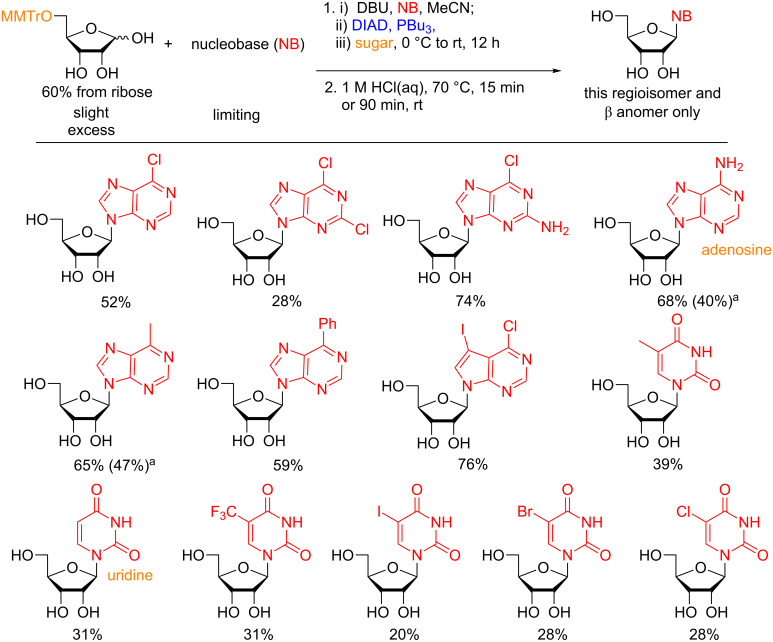
Direct synthesis of furanosyl nucleosides from 5-*O*-monoprotected ribose in a one-pot glycosylation–deprotection strategy. ^a^Yield in parentheses is after crystallization from MeOH due to trace impurities still present after chromatographical purification. DIAD = diisopropyl azodicarboxylate.

Although this route represents the first neutral protecting-group-free glycosylation of heterocycles, in most instances the furanosyl nucleoside is desired as the product. Therefore, we utilized a monoprotection strategy where an acid-labile protecting group was installed regioselectively in one step at C5 of ribose, hence locking the compound in the furanose conformation. Subsequently, the glycosylation was performed followed by cleavage of the protecting group using aqueous mineral acid in one pot. This route exclusively afforded the β-anomer in moderate to good yields. As in the case of the pyranosyl ribonucleosides, the purines proved more reactive than the pyrimidines (yields shown in Figure 10). In many instances the nucleoside is accessible in the highest overall yield ever reported. Additionally, adenosine and uridine are accessible by these conditions.

**Figure 11:**
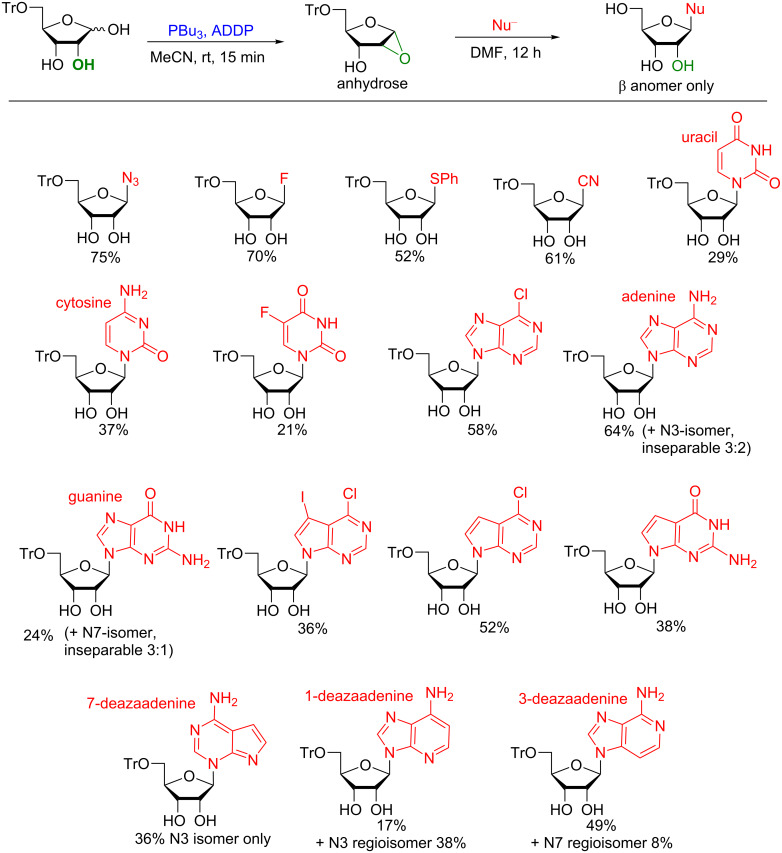
Synthesis of ribofuranosides using a monoprotected ribosyl donor via an anhydrose intermediate.

Realizing the tremendous potential of this reaction, we carried out an in-depth study to further expand the substrate scope and to evaluate the mechanism [108]. Knowing that the furanoside is only obtainable in appreciable amounts using a 5-*O*-monoprotected ribosyl donor, we utilized 5-*O-*tritylribose in this study. By slightly modifying the molar equivalents and order of addition of the reagents and by utilizing the electron acceptor 1,1'-(azodicarbonyl)dipiperidine (ADDP) instead of DIAD, we could observe by NMR the in situ formation of a 1,2-anhydrosugar, termed anhydrase. This compound was stable in the absence of moisture for more than one month (Figure 11). The epoxide could then be subjected to nucleophilic ring opening by small nucleophiles (-N_3_, -CN, -SPh, -F) or deprotonated nucleobases. These conditions proved to be much more general allowing for the synthesis of many nucleosides that were not available in the first communication. These included glycosylation of all four natural nucleobases found in ordinary RNA (uracil, cytidine, adenine, and guanine) as well 7-deazapurines unsubstituted at position 7. However one major drawback of the method was found to be the poor regiochemical control observed with the adenine scaffold. The monoprotected adenosine analog was isolated as a 3:2 N9/N3 mixture (using purine numbering) in reasonable yield. When repeating the reaction with 7-deazaadenine as the substrate only the N3 regioisomer was observed in poor yield. The same regioisomer also was the major product when 1-deazaadenine was used as the starting material. When 3-deazaadenine was used, finally the desired N9 regioisomer was formed (49%) accompanied by only traces of the N7 regioisomer.

To our delight, all of the tritylated nucleosides having the biologically relevant (N1 for pyrimidines, N9 for purines) regioisomeric geometry could be deprotected using aqueous TFA in a similar one-pot sequence to the preliminary work (not shown).

**Figure 12:**
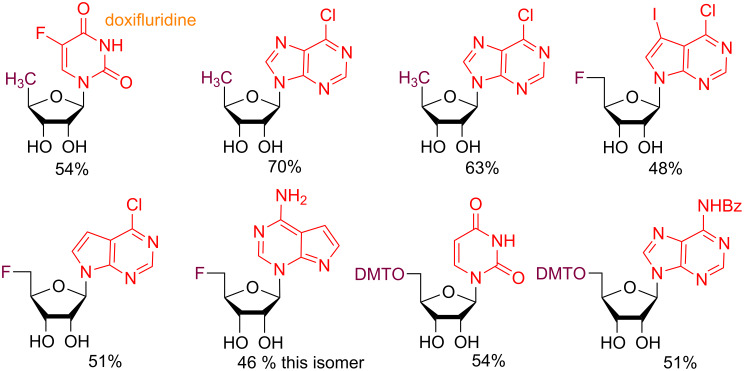
C5′-modified nucleosides available under our conditions.

We also demonstrated the amenability of these conditions to other furanoses, namely 5-deoxyribose, 5-deoxy-5-fluororibose, and 5-*O*-dimethoxytritylribose donors (Figure 12), which are important in medicine [109] and medicinal chemistry [110], or solid-phase automated phosphoramidite oligonucleotide synthesis [111], respectively. Briefly, the anticancer prodrug doxifluridine [109] was available under these conditions in moderate yield (54%) as was a N6-benzoylated adenosine analog (51% yield) regioselective for the N9 isomer.

**Scheme 40:**
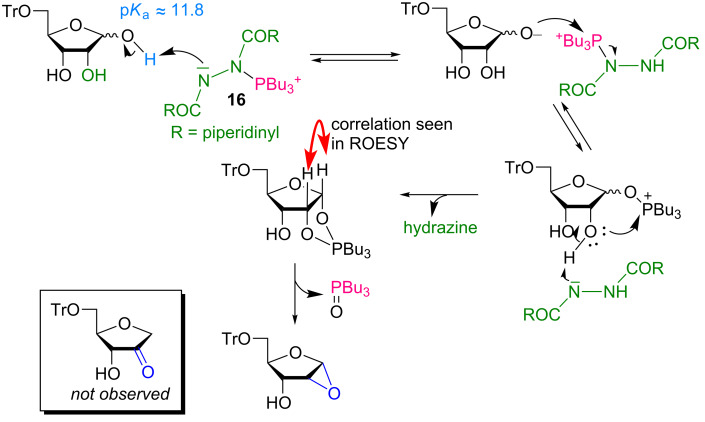
Plausible reaction mechanism for the formation of the anhydrose.

As a result of this very powerful, albeit unusual, reactivity we undertook a mechanistic investigation to ascertain the reason for the formation of the epoxide as well as to rationalize why it is the only product formed (Scheme 40). Mitsunobu reactions with diols have been shown to proceed through a 5-membered 1,3λ-dioxaphospholane intermediate [112–114], which then extrudes phosphine oxide resulting in the epoxide. With P(*n-*Bu)_3_, a highly reactive phosphine such as phosphorane has been observed only twice before when the simple substrate ethylene glycol was used as the diol [115–116]. Operating under cryogenic conditions, we were able to observe the phosphonium betaine as well as the dioxaphospholane intermediate and confirmed its 1,2-*cis* stereochemistry by NMR. The rearrangement of the dioxaphospholane to the anhydrose was modeled by density functional theory (DFT) calculations at the level of B3LYP-D3/6-311+G**. Interestingly, it was found that the energy barrier for the formation of the anhydrose was 17.5 kcal mol^−1^. The alternative reaction pathway leading to the thermodynamically favored 3-tetrahydrofuranone derivative proceeds over the energy barrier of 19.8 kcal mol^−1^ which is too high and is not observed, hence explaining the excellent selectivity toward the formation of the anhydrose. This work showcases a new method for accessing biologically relevant nucleosides and the procedure is general allowing access to other aglycones. However, the major drawback is certainly the low regioselectivity observed for some purine substrates, especially those containing an electron-donating group at position 6 of the ring.

#### 5.2 Catalytic conditions

Perhaps most fascinating is the fact that catalytic conditions have now been elucidated to produce glycosides from unprotected sugars. We do note, though, that to date, only simple alcohols can be glycosylated under catalytic conditions and no mechanism has been determined yet. However, we are optimistic that further progress in this area will be made very soon.

Two promising catalytic protecting-group-free strategies have been explored by the Mahrwald group. In their first study, they devised metal-catalyzed conditions to obtain aliphatic pentosides where the biologically relevant furanoside was the major product. Their second study dealt with organocatalyzed reactions and provided either the pyranoside or furanoside as the major product depending on the conditions.

**Figure 13:**
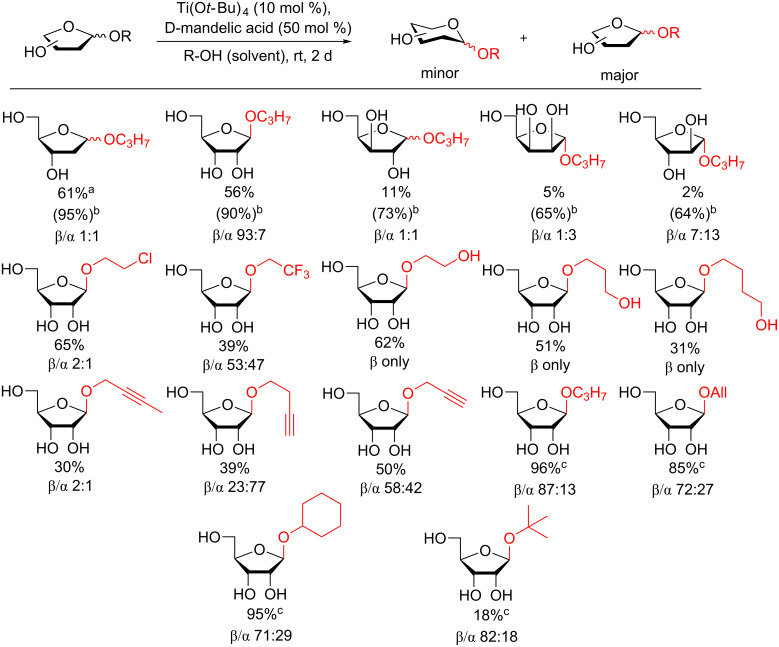
Direct glycosylation of several aliphatic alcohols using catalytic Ti(O*t-*Bu)_4_ in the presence of D-mandelic acid. The furanoside is the major or exclusive product. ^a^Only 4 mol % D-mandelic acid used. ^b^After 12 days and 1.0 equiv LiBr added. ^c^1.0 equiv LiBr added to enhance the yield.

Using Ti(II)-catalyzed conditions in the presence of D-mandelic acid, the glycosyl furanosyl aliphatic alcohol could be obtained as the major product and the results are summarized in Figure 13 [117]. The yields ranged from very moderate to nearly quantitative and the reaction was reasonably stereoselective for the 1,2-*trans* product. The disadvantages of this otherwise extremely attractive procedure are the long reaction time and the fact that the alcohol had to be used as solvent. This severely limits its potential as a method to synthesize di- or oligosaccharides or other biologically important molecules.

**Figure 14:**
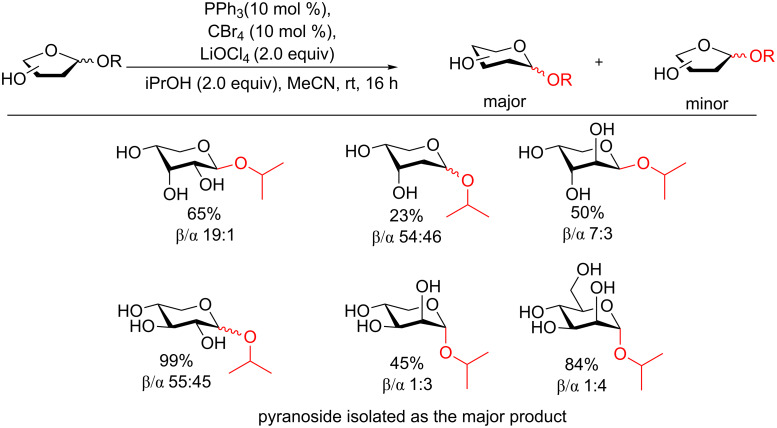
Access to glycosides using catalytic PPh_3_ and CBr_4_.

In 2013 a follow up study Schmalisch and Mahrwald developed an even further simplified catalytic procedure (Ph_3_P and CBr_4_) with a larger substrate scope to provide both aliphatic and benzylic alcohols in moderate to good yields [118]. In the first part of this study isopropanol was used only in moderate excess and MeCN as a solvent. The authors also reported that the yield could be augmented by the use of LiOCl_4_. It is known that Li^+^ salts are promoters of glycosylation reactions [119] and these conditions provided the isopropyl pyranosides as the major product. As in the previous study there was moderate to good stereoselectivity for the 1,2-*trans* glycoside (Figure 14).

**Figure 15:**
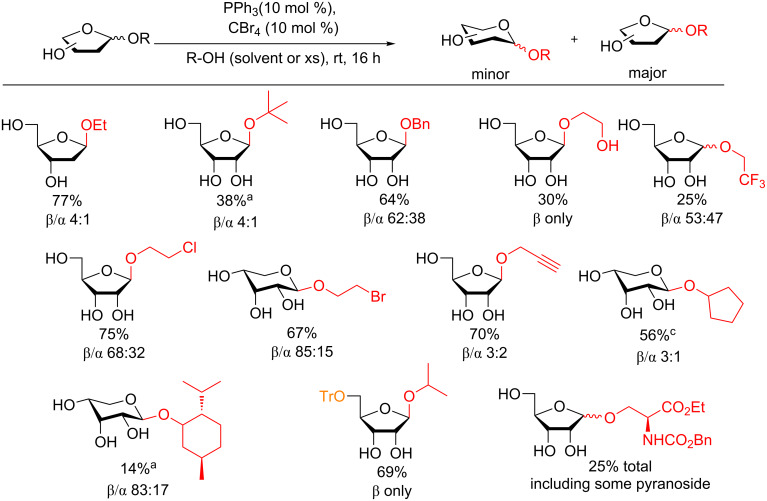
Access to ribofuranosyl glycosides as the major product under catalytic conditions. ^a^LiOCl_4_ (2.0 equiv.) was used to bolster the yield.

In the second part of the study, various alcohol donors were employed, however, they were either used as solvent or in large excess. Herewith the more attractive furanosides presented as the major products (Figure 15). The authors demonstrated that the reaction proceeds under neutral conditions by using the acid-sensitive 5-*O*-tritylribose as a donor and that the reaction is not driven by triphenylphosphine oxide formation. In very moderate yield the authors could even form an O-linked glycosyl bond as a mixture of diastereomers with a protected serine moiety under their conditions further exploring the potential of this reaction.

These two catalytic studies are tremendous developments in carbohydrate synthesis. It is very encouraging to now have access to glycosides fully devoid of protecting groups under simple catalytic conditions. We must note that these examples include only simple alcohols as the acceptor, but we remain optimistic that higher alcohols and other saccharides may be studied in the future.

## Conclusion

We have presented an overview of modern protecting-group-free glycosylation strategies with mechanistic rationale, when available, to account for the regiochemical activation at the anomeric position. The protecting-group-free glycosylation at first glance looks like a nearly impossible task, but the inherent different reactivity of the reducing end of a saccharide unit is fascinating, and even more interesting is the creative ways modern chemists are exploiting it. It is truly groundbreaking that Lewis acid-mediated [56–57] and transition-metal-catalyzed [62,65] strategies are feasible even with other reactive groups present on the donor molecule and that access to the always challenging 1,2-*cis* glycosides are available by employing a remote activation strategy [42–43,49–50]. We acknowledge the incredibly operationally simple C3′–OH regioselective glycosylation of sucrose as particularly fascinating [57]. Of course, these methods are setback by the need to use protecting groups to furnish the reactive donor species, but we are optimistic more streamlined approaches are forthcoming.

The creative ways to activate the anomeric center directly, from an unprotected glycoside are truly captivating and the efforts of the Shoda, Fairbanks, Nitz, and other groups are major breakthroughs. What is further enticing is the ability for nearly all of the direct activation strategies to take place in aqueous medium. This is environmentally friendly and perfectly amenable to the application in chemical biology as well. Particularly exciting developments include the protecting-group-free ligation of saccharides to proteins as well as access to isolatable oxazolines as substrates for endoglycosidases [69,72]. Because the substrate scope is somewhat limited to the glycosylation of reasonably simple molecules, with the exception of those employed in enzymatic glycosylation, we hope that more complex molecules (such as O*-* and N-linked glycans) will be available in the near future. We postulate that by combining some of the activation strategies of section 4 with some of the Lewis acid-mediated or transition-metal-catalyzed methods developed in section 3 the access to more intricate products will be facilitated. While numbers of methods are available for the chemoselective activation of the anomeric position, much less attention has been paid to the regioselective reactivity of the nucleophiles, in particular of the different OH groups of sugar acceptors.

One of the most important tools added to the organic chemist’s toolbox in the last 50 years has been the Mitsunobu reaction and we are delighted to see that such a simple reaction can now be employed for the synthesis of glycosides using fully or minimally protected saccharide donors due to the lowered p*K*_a_ of the anomeric proton. Of particular note is the ability to synthesize biologically active nucleosides in one pot from a monoprotected ribosyl moiety [107] and that a full mechanistic rationale is now available to account for this unique reactivity [108]. The most obvious improvement is to await a method that provides furanosyl glycosides in preference to pyranosides when furanose donors are employed, thus obviating the need for any protecting groups. We also credit the outstanding efforts of the Mahrwald group for being able to develop a catalytic methodology for the synthesis of aliphatic or benzylic glycosides under neutral conditions [117–118]. We are looking forward to seeing if any expansion of the substrate scope to more complex acceptors is possible and forthcoming.

Although the foundation for this powerful chemistry was laid over 100 years ago with the Fisher glycosylation, numerous obstacles had to be overcome when designing novel methods in this field. However, the challenge to obtain regio- and stereoselective products in the presence of other nucleophilic groups on the donor molecules will always persist. As it can be seen by the number of publications in the last couple of years this field is of ongoing scientific interest and we are looking forward to upcoming results.
